# Effects of a Responsive Parenting Intervention Among Black Families on Infant Sleep: A Secondary Analysis of the Sleep SAAF Randomized Clinical Trial

**DOI:** 10.1001/jamanetworkopen.2023.6276

**Published:** 2023-03-31

**Authors:** Justin A. Lavner, Emily E. Hohman, Steven R. H. Beach, Brian K. Stansfield, Jennifer S. Savage

**Affiliations:** 1 Department of Psychology, University of Georgia, Athens; 2 Center for Childhood Obesity Research, The Pennsylvania State University, University Park; 3 Center for Family Research, University of Georgia, Athens; 4 Department of Pediatrics, Augusta University, Augusta, Georgia; 5 Department of Nutritional Sciences, The Pennsylvania State University, University Park

## Abstract

**Importance:**

Black individuals in the US experience sleep disparities beginning in infancy and continuing throughout the lifespan, suggesting early interventions are needed to improve sleep.

**Objective:**

To investigate whether a responsive parenting (RP) intervention for Black mothers improves infant sleep and increases responsive sleep parenting practices.

**Design, Setting, and Participants:**

This is a post hoc secondary analysis of the Sleep SAAF (Strong African American Families) study, a randomized clinical trial comparing an RP intervention with a safety control condition over the first 16 weeks post partum. Data were collected between spring 2018 and summer 2021. Families were recruited from the mother-infant nursery at Augusta University Medical Center, Augusta, Georgia, and completed home visits at 1, 3, 8, and 16 weeks post partum. Primiparous Black mother-infant dyads were screened for eligibility using medical records. Results were analyzed on an intention-to-treat model. Data were analyzed from March 2022 to January 2023.

**Interventions:**

The RP intervention curriculum focused on infant sleep, soothing and crying, and feeding. The control group received a safety intervention. Community research associates delivered the interventions during home visits at 3 and 8 weeks post partum.

**Main Outcomes and Measures:**

The primary end point was infant sleep duration at 16 weeks. Mothers reported on bedtime routine and sleep behaviors, infant sleep duration, and nighttime waking and feeding at 8 and 16 weeks post partum using questionnaires.

**Results:**

A total of 212 Black mothers (mean [SD] age, 22.7 [4.5] years) were randomized, including 208 mothers (98.6%) who identified as non-Hispanic and 3 mothers (1.4%) who identified as Hispanic; 108 mothers were randomized to the RP group and 104 mothers were randomized to the control group. At 16 weeks post partum, infants in the RP group had longer reported nighttime sleep duration (mean difference, 40 [95% CI, 3 to 77] minutes), longer total sleep duration (mean difference, 73 [95% CI, 14 to 131] minutes), fewer nighttime wakings (mean difference, −0.4 [95% CI, −0.6 to −0.1] wakings), and greater likelihood of meeting guidelines of at least 12 hours of total sleep per day (risk ratio [RR], 1.4 [95% CI, 95% CI, 1.1 to 1.8]) than controls. Relative to controls, mothers in the RP group more frequently reported engaging in some RP practices, including giving the baby a few minutes to fall back asleep on their own (RR, 1.6 [95% CI, 1.0 to 2.6]) and being less likely to feed their baby as the last activity before bed (RR, 0.5 [95% CI, 0.3 to 0.8]).

**Conclusions and Relevance:**

This secondary analysis of a randomized clinical trial found that an RP intervention for Black families improved infant sleep and increased some responsive sleep parenting practices.

**Trial Registration:**

ClinicalTrials.gov Identifier: NCT03505203

## Key Points

**Question:**

Does a responsive parenting intervention for Black families improve infant sleep?

**Findings:**

In this secondary analysis of a randomized clinical trial of 194 Black mother-infant dyads, infants in the responsive parenting group had significantly longer nighttime sleep duration, longer total sleep duration, fewer night wakings, and were more likely to meet recommended guidelines of at least 12 hours of sleep per day at age 16 weeks than the control group.

**Meaning:**

These findings suggest that responsive parenting interventions delivered early in infancy can improve sleep among Black infants and may be an effective approach to reducing sleep and sleep-related disparities early in the lifespan.

## Teaser

This secondary analysis of the Sleep SAAF (Strong African American Families) randomized clinical trial investigates whether a responsive parenting intervention improves infant sleep and responsive parenting practices among Black families in the US.

## Introduction

Black individuals in the US experience disparities in sleep throughout their lifespan.^1,2,3^ During infancy, Black infants have shorter sleep durations than White infants and are less likely to meet recommended guidelines of at least 12 hours of sleep per day.^4,5,6^ These patterns are concerning, given links between infants’ sleep and later outcomes, including child overweight and obesity,^7,8^ social-emotional functioning,^9,10^ and cognitive development.^11,12^ Behavioral interventions for parents can improve infants’ sleep by encouraging the use of recommended parenting practices, such as consistent bedtime routines and developmentally appropriate responses to night wakings.^13,14,15,16,17,18^ These interventions are seen as a promising approach to reducing sleep and sleep-related health disparities early in development.^19,20^ To date, however, implementation of these interventions among racially and ethnically diverse populations has been limited.^21^ To our knowledge, only 1 intervention study has specifically aimed to promote better sleep among Black infants,^22^ and results from that study indicated no significant differences between the intervention and control groups in maternal reports of infant sleep problems or infant nighttime awakenings over the first 15 months post partum.^23^ As such, there is a need to identify behavioral interventions that improve infant sleep among Black families.

To address this gap, we designed the Sleep SAAF (Strong African American Families) study for primiparous Black mothers and their newborn infants.^24^ This 2-group randomized clinical trial tested the effects of a responsive parenting (RP) intervention focused on promoting infant sleep and self-soothing relative to a safety control over the first 16 weeks post partum to reduce rapid infant weight gain. The Sleep SAAF RP intervention was adapted from the Intervention Nurses Start Infants Growing on Healthy Trajectories (INSIGHT) RP intervention, a nurse-delivered curriculum that provided primiparous mothers with guidance on infant sleep, feeding, soothing, and interactive play.^25^ The INSIGHT RP sleep curriculum included messaging on bedtime routines, sleep location and behaviors, and night waking.^26^ Among the predominantly White, middle-class sample of first-time mothers and their infants, INSIGHT RP infants had longer nighttime sleep duration at ages 8 weeks (35 minutes), 16 weeks (25 minutes), and 40 weeks (22 minutes) relative to controls; were more likely to self-soothe to sleep; and were less likely to be fed immediately before bed or back to sleep after night wakings.^26^

In this post hoc secondary analysis from the Sleep SAAF trial, we examine effects of the Sleep SAAF RP intervention on infant sleep duration and responsive parenting sleep behaviors, building on our earlier work examining intervention effects on rapid infant weight gain,^27^ feeding practices,^28^ and maternal responses to infant distress.^29^ We hypothesized that there would be longer infant sleep duration and more positive sleep behaviors (bedtime routines, responses to night wakings) among RP families relative to controls.

## Methods

**Figure 1.**
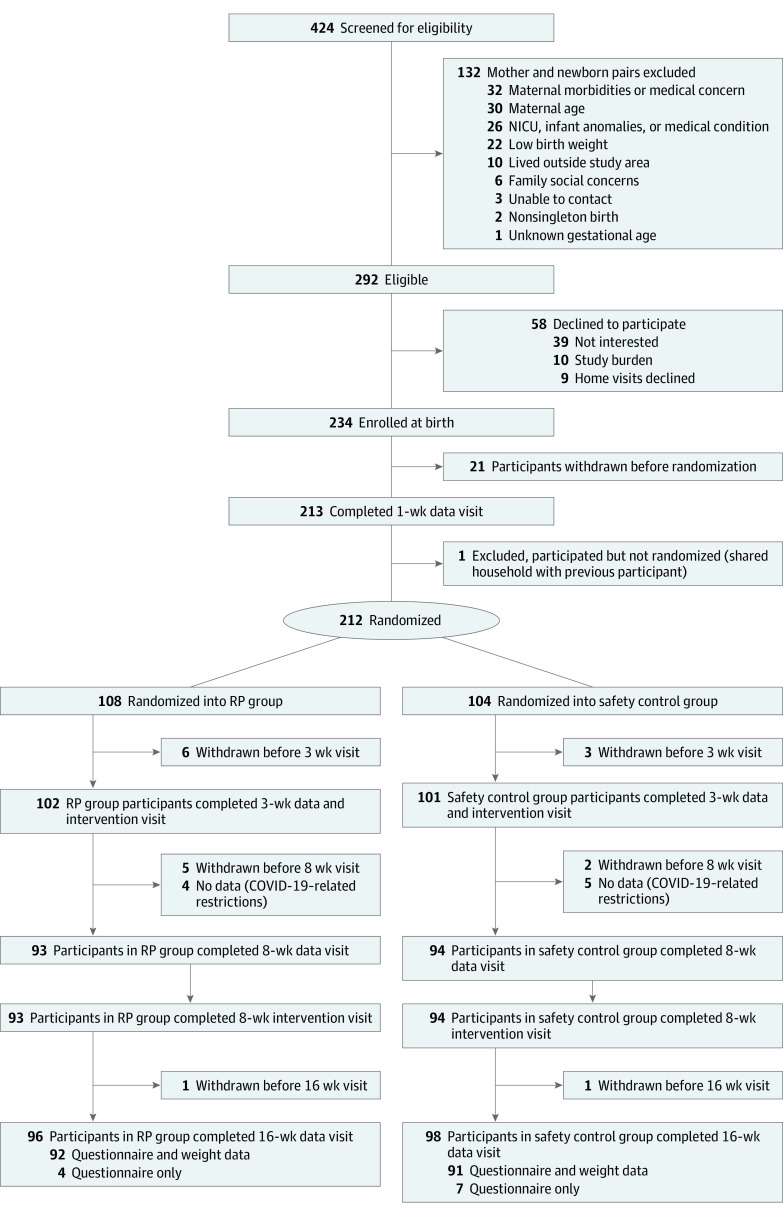
Study Recruitment Flowchart NICU indicates neonatal intensive care unit; RP, responsive parenting.

The Sleep SAAF study was approved by the Augusta University institutional review board. Mothers provided written informed consent for participation in the hospital at the time of recruitment. This study followed the Consolidated Standards of Reporting Trials (CONSORT) reporting guideline for randomized studies (Figure 1). Additional details on the study design have been published previously^24,27^; the trial protocol and statistical analysis plan are provided in Supplement 1.

### Participants and Study Design

Mother-infant dyads were recruited shortly after delivery from the mother-infant nursery at Augusta University Medical Center in Augusta, Georgia. Recruitment spanned spring 2018 through spring 2021, with a pause from March 9 to August 31, 2020, due to the COVID-19 pandemic. Mothers were eligible if they were primiparous, aged at least 17 years, self-identified as Black or African American, spoke English, and lived within 75 miles of Augusta, Georgia. Eligible infants were singleton, had a gestational age of at least 37 weeks, and weighed at least 2500 g at birth. Dyads were excluded if the mother had a known medical condition that could impact postnatal care (eg, serious mental illness, substance use disorder), the infant had a medical condition impacting feeding or growth (eg, cleft palate), there was an adoption plan in place, or there was a plan to move out of the area within 4 months of delivery. Ethnicity was self-reported as Hispanic or non-Hispanic. Ethnicity data were collected to characterize the cohort.

Mother-infant dyads received home visits from trained community research associates (CRAs) at approximately 1, 3, 8, and 16 weeks post partum. To minimize bias during the assessments, separate CRAs served as data collectors (at 1, 8, and 16 weeks) and intervention facilitators (at 3 and 8 weeks). Furthermore, intervention facilitators were trained in (and subsequently delivered) only 1 of the 2 study interventions (RP or safety control). CRAs and study participants were unaware of which condition was the experimental condition. Participating mothers received $50 after the 1-week visit, $50 after the 3-week visit, $100 after the 8-week visit, and $100 after the 16-week visit.

Participants were randomized to receive the RP intervention or the child safety control intervention after the 1-week data collection visit. Randomization was stratified by sex-specific birth weight for gestational age (<50th percentile or >50th percentile) and intended feeding mode (breastfeeding or formula). Randomization was performed using a secure Microsoft Excel application prepared by a statistician unaffiliated with the study and administered by the study’s project coordinator.

### Study Interventions

Intervention curricula were delivered by CRAs at the 3- and 8-week home visits. In both conditions, mothers were provided with a packet of intervention handouts that the facilitators talked through with the mother, supplemented with hands-on activities, discussion, and videos. Methods and intervention materials for both groups were tailored for Black families.^24,27^

Details on the Sleep SAAF curriculum are published elsewhere.^24,27^ In brief, the RP curriculum focused on infant sleep, soothing and crying, feeding, and interactive play during the first months after birth. All topics were discussed in detail at the 3-week visit (which took approximately 90-120 minutes), and the same material was reviewed at the 8-week visit (which took approximately 45-60 minutes). Regarding sleep, guidance included information about normal sleep patterns in infants, including how many hours of sleep infants typically need, frequency of night waking, napping, and tiredness cues (eg, yawning, fixed stare, eye rubbing). Mothers were also taught to establish a consistent bedtime routine that included putting the infant to bed early (ideally by 8 pm) and that would help the baby be calm, comfortable, relaxed, and drowsy but awake so that they would learn to fall asleep on their own. Mothers were encouraged to avoid feeding the infant to sleep or putting the infant to bed with a bottle, and were provided with information regarding so-called *dream feeds*, in which the mother wakes the baby to feed before the mother goes to bed. Safe sleep strategies to prevent sudden infant death syndrome (SIDS) were also discussed, including always placing baby on their back on a firm sleep surface and avoiding the use of pillows and blankets. Mothers also received messaging on responding appropriately when their baby cries at night, including giving baby a couple of minutes to fall back asleep on their own, using quiet calming messages (eg, shushing, white noise) before picking baby up, only feeding if baby shows signs of hunger, and putting baby back down drowsy but awake.

The control curriculum was matched for intensity and session length but focused on sleep and home safety. CRAs taught mothers about SIDS facts and myths, reducing baby’s risk of SIDS (eg, placing baby on their back on a firm sleep surface; keeping baby’s sleep area close to, but separate from, where the mother sleeps; using a pacifier), the importance of good health care, taking care of a crying baby (eg, preventing shaken baby syndrome), finding caretakers for baby, and food safety.

### Measures

Demographic information was collected from mothers at 1 week post partum, and child sex, gestational age, and birthweight were abstracted from medical records. At infant ages 3, 8, and 16 weeks, mothers completed the Brief Infant Sleep Questionnaire (BISQ)^30^ via an online survey platform (Qualtrics). The BISQ assesses infant sleep environment, bedtime routine activities, sleep duration, and parental response to nighttime awakenings. Items that were developmentally appropriate were included. The 3- and 8-week questionnaires were identical, and additional items were included at 16 weeks. To assess the main outcome, infant sleep duration at 16 weeks, mothers reported on total nighttime (7 pm-8 am) and daytime (8 am-7 pm) sleep in hours. Five items on safe sleep practices, such as sleep position and location,^31^ were also collected at 16 weeks. All items are reported in the eMethods in Supplement 2.

### Statistical Analysis

Analyses were performed in SAS statistical software version 9.4 (SAS Institute). Descriptive statistics were generated, and differences between groups are presented as the difference between group means or relative risk ratios (RRs) with 95% CIs. Per the study protocol,^24^ statistical tests of intervention effects at 8 and 16 weeks were conducted using mixed-effects models, controlling for concurrent infant weight-for-age *z*-score. Intervention group and group-by-time effects were tested. The mixed-effects models use full-information maximum likelihood to handle missing data. For outcomes that were only collected at 16 weeks, linear or logistic regression, controlling for weight-for-age *z*-score, were performed. A subset of 11 participants provided sleep data at 16 weeks but did not have infant weight data collected at this time point due to COVID-19–related restrictions on in-person measurement. These data were treated as missing completely at random and subject to listwise deletion in the linear and logistic models. Statistical significance was defined as 2-tailed *P* < .05. As these are secondary analyses, no adjustment for potential Type I error due to multiple tests was made. Thus, these results should be considered exploratory.

Statistical power for the study was originally calculated for the primary outcome of between-group differences in conditional weight gain scores from 3 to 16 weeks, which is reported elsewhere.^27^ Target enrollment was 300 mother-infant dyads based on the INSIGHT effect size of approximately 0.4 for infant conditional weight gain at 6 months.^32^ Enrollment ended in spring 2021 before this target was met due to challenges from the COVID-19 pandemic and the funding timeline. Data were analyzed from March 2022 to January 2023.

## Results

A total of 212 mothers (mean [SD] age, 22.7 [4.5] years) were analyzed, including 208 mothers (98.6%) who identified as non-Hispanic and 3 mothers (1.4%) who identified as Hispanic; 108 mothers were randomized to the RP group and 104 mothers were randomized to the control group. Table 1 presents family demographics. Most mothers had some high school or completed high school (132 mothers [62.3%]). Descriptive statistics for sleep outcomes at the 3-week preintervention baseline are provided in the eTable in Supplement 2.

**Table 1.** Participant Demographics

Characteristic	Participants, No. (%)		
	Total (N = 212)	RP (n = 108)	Control (n = 104)
**Mothers**			
Age, mean (SD), y	22.7 (4.5)	23.4 (4.9)	22.0 (3.9)
Prepregnancy BMI, mean (SD)	28.1 (8.2)	27.8 (8.5)	28.4 (7.9)
Ethnicity			
Hispanic	3 (1.4)	2 (1.8)	1 (1.0)
Non-Hispanic	208 (98.6)	106 (98.2)	102 (99.0)
Romantic status			
Single	86 (40.5)	42 (38.9)	44 (42.3)
Married and living together	22 (10.4)	15 (13.9)	7 (6.7)
Married but not living together	0	0	0
Not married and living together	66 (31.1)	33 (30.6)	33 (31.7)
Involved in a steady relationship but not living together	37 (17.5)	17 (15.7)	20 (19.2)
Involved in an on-off again relationship	1 (0.5)	1 (0.9)	0
Annual household income, $			
<10 000	48 (22.7)	28 (26.2)	20 (19.2)
10 000-24 999	25 (11.8)	8 (7.5)	17 (16.4)
25 000-49 999	29 (13.7)	19 (17.8)	10 (9.6)
≥50 000	16 (7.6)	8 (7.5)	8 (7.7)
Do not know	84 (39.8)	38 (35.5)	46 (44.2)
Refuse to answer	9 (4.3)	6 (5.6)	3 (2.9)
Education			
Some high school	30 (14.2)	15 (13.9)	15 (14.4)
High school graduate	102 (48.1)	50 (46.3)	52 (50.0)
Some college or technical school	53 (25.0)	27 (25.0)	26 (25.0)
Completed college	19 (9.0)	10 (9.3)	9 (8.7)
Post graduate training or degree	8 (3.8)	6 (5.6)	2 (1.9)
Received federal Nutrition Assistance			
SNAP	101 (49.0)	53 (50.5)	48 (47.5)
WIC	159 (76.4)	84 (80.0)	75 (72.8)
**Infants**			
Gestational age, mean (SD), wk	39.1 (1.0)	39.1 (1.1)	39.1 (1.0)
Enrollment weight, mean (SD), kg	3.02 (0.37)	3.06 (0.38)	2.99 (0.36)
Sex			
Female	110 (51.2)	55 (50.9)	55 (52.9)
Male	102 (48.1)	53 (49.1)	49 (47.1)
Breastfed (any)			
At 8 wk	65 (34.8)	36 (38.7)	29 (30.9)
At 16 wk	43 (22.2)	22 (22.9)	21 (21.4)

Abbreviations: BMI, body mass index (calculated as weight in kilograms divided by height in meters squared); RP, responsive parenting; SNAP, Supplemental Nutrition Assistance Program; WIC, Special Supplemental Nutrition Program for Women, Infants, and Children.

### Sleep Duration

**Figure 2.**
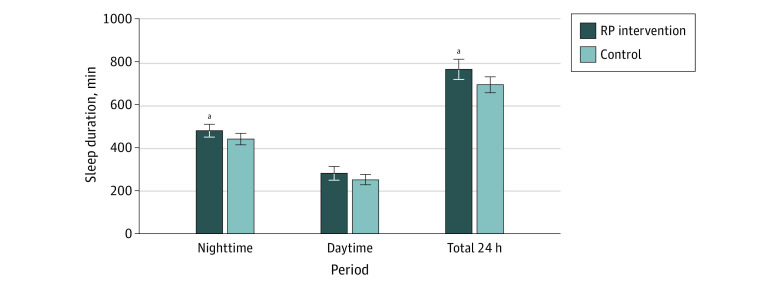
Nighttime, Daytime, and Total 24-h Sleep Duration at Infant Age 16 Weeks RP indicates responsive parenting; error bars, SD. Nighttime was defined as 7 pm to 8 am; daytime, 8 am to 7 pm. ^a^Statistically significant difference.

At 16 weeks, infants in the RP group had longer reported nighttime sleep duration than controls, with a mean difference of 40 (95% CI, 3 to 77) minutes (Table 2, Figure 2). RP infants also slept a mean of 31 (95% CI, −7 to 69) minutes longer during the day, although this difference was not statistically significant. Combining nighttime and daytime sleep, RP infants slept a mean of 73 (95% CI, 14 to 131) minutes longer over the entire 24 hours than controls. This difference translated into more RP infants achieving the minimum sleep duration of 12 hours per day recommended for infants aged 4 to 11 months^33,34^ than controls (61 infants [64.9%] vs 44 infants [45.8%]; RR, 1.4 [95% CI, 1.1 to 1.9]).

**Table 2.** Bedtime Routines and Sleep Behaviors

Measure	8 wk			16 wk			Model *P* value^a^	
	No. (%)		Estimate (95% CI)^b^	No. (%)		Estimate (95% CI)^b^	Group	Group × time
	RP (n = 93)	Control (n = 94)		RP (n = 96)	Control (n = 98)			
Sleep duration, mean (SD) min								
Night (7 pm-8 am)	NA	NA	NA	481 (135)^c^	441 (126)^d^	40 (3 to 77)^e^	.02	NA
Day (8 am-7 pm)	NA	NA	NA	283 (147)^f^	251 (118)^g^	31 (−7 to 69)^e^	.10	NA
Total	NA	NA	NA	765 (223)^f^	693 (182)^g^	73 (14 to 131)^e^	.009	NA
Meets recommended ≥12 h sleep	NA	NA	NA	61 (64.9)^f^	44 (45.8)^g^	1.4 (1.1 to 1.8)^h^	.002	NA
Time in bed, mean (SD), min	621 (104)^i^	582 (108)^j^	42 (10 to 74)^e^	635 (106)^k^	622 (100)^g^	10 (−21 to 40)^e^	.05	.07
Has a bedtime routine	81 (87.1)	62 (66.7)^k^	1.3 (1.1 to 1.5)^h^	85 (88.5)	80 (81.6)	1.1 (1.0 to 1.2)^h^	.004	.42
Fed as last activity before bed	25 (26.9)	33 (35.1)	0.8 (0.5 to 1.2)^h^	18 (18.8)	37 (37.8)	0.5 (0.3 to 0.8)^h^	.01	.12
Bedtime 8 pm or earlier	27 (29.7)^i^	18 (20.5)^l^	1.5 (0.9 to 2.4)^h^	23 (24.7)^k^	25 (25.5)	1.0 (0.6 to 1.6)^h^	.35	.25
Falls asleep swaddled	12 (12.9)	11 (11.7)	1.1 (0.5 to 2.4)^h^	5 (5.2)	3 (3.1)	1.7 (0.4 to 6.9)^h^	.48	.49
Falls asleep with pacifier	22 (23.7)	18 (19.2)	1.2 (0.7 to 2.1)^h^	31 (32.3)	27 (27.6)	1.2 (0.8 to 1.8)^h^	.28	.98
Falls asleep with white noise	21 (22.6)	8 (8.5)	2.7 (1.2 to 5.7)^h^	17 (17.7)	5 (5.1)	3.5 (1.3 to 9.0)^h^	.001	.81
Falls asleep being held	28 (30.1)	30 (31.9)	0.9 (0.6 to 1.4)^h^	27 (28.1)	27 (27.6)	1.0 (0.6 to 1.6)^h^	.99	.71
Dream feeds	66 (72.5)^i^	37 (39.8)^k^	1.8 (1.4 to 2.4)^h^	60 (62.5)	42 (43.3)^d^	1.4 (1.1 to 1.9)^h^	<.001	.07
Usually or always put to bed awake^m^	19 (20.4)	9 (9.7)^k^	2.1 (1.0 to 4.4)^h^	22 (23.2)^c^	13 (13.3)	1.7 (0.9 to 3.3)^h^	.02	.68
Time to fall asleep, min	NA	NA	NA	17.9 (9.9)^f^	20.4 (12.6)^g^	−2.6 (−5.9 to 0.7)^e^	.17	NA
Put to sleep on back	78 (86.7)^n^	88 (93.6)	0.9 (0.8 to 1.0)^h^	78 (81.3)	88 (90.7)^d^	0.9 (0.8 to 1.0)^h^	.05	.76
Sleeps in own crib or bassinet								
Usually	81 (87.1)	72 (76.6)	1.1 (1.0 to 1.3)^h^	79 (82.3)	77 (78.6)	1.0 (0.9 to 1.2)^h^	.15	.16
Always^o^	NA	NA	NA	42 (43.8)	32 (33.0)^d^	1.3 (0.9 to 1.9)^h^	.05	NA
Never sleeps with toys or stuffed animals^o^	NA	NA	NA	85 (88.5)	78 (81.3)^g^	1.1 (1.0 to 1.2)^h^	.18	NA
Never sleeps in same bed as adult or another child^o^	NA	NA	NA	41 (42.7)	27 (27.8)^d^	1.5 (1.0 to 2.3)^h^	.01	NA
Always place baby on back for sleep^o^	NA	NA	NA	64 (67.4)^c^	71 (73.2)^d^	0.9 (0.8 to 1.1)^h^	.40	NA
Never sleeps on sofa, waterbed, soft mattress^o^	NA	NA	NA	69 (71.9)	64 (66.0)^d^	1.1 (0.9 to 1.3)^h^	.22	NA

Abbreviations: NA, not applicable or not assessed at this age; RP, responsive parenting.

^a^ *P* values are from generalized linear models, adjusting for infant weight-for-age *z*-score. These models exclude 11 participants for whom weight was not collected at 16 weeks due to COVID-19 pandemic restrictions.

^b^ Unadjusted, includes all available data.

^c^ Among 95 participants.

^d^ Among 97 participants.

^e^ Expressed as mean difference.

^f^ Among 94 participants.

^g^ Among 96 participants.

^h^ Expressed as risk ratio.

^i^ Among 91 participants.

^j^ Among 84 participants.

^k^ Among 93 participants.

^l^ Among 88 participants.

^m^ Response options were never, sometimes, usually, or always.

^n^ Among 90 participants.

^o^ Response options were always, usually, sometimes, occasionally, or never.

### Bedtime Routine and Sleep Behaviors

At 8 weeks, RP group infants spent a mean of 42 (95% CI, 10 to 74) minutes longer in bed than control infants, but there was no group difference in time in bed at 16 weeks (Table 2). At 8 weeks, RP infants had earlier mean bedtimes than controls (8:53 pm vs 9:20 pm; mean difference, 27 [95% CI, 7 to 47] minutes). At 16 weeks, bedtimes did not differ between groups (9:00 pm vs 9:01 pm; mean difference, 1 [95% CI, −18 to 21] minutes), and there was no group difference at 8 or 16 weeks in the percentage of infants with a bedtime of 8 pm or earlier, as recommended in the RP intervention. Regarding bedtime routines, RP infants were more likely than controls to have a bedtime routine at 8 weeks (81 infants [87.1%] vs 62 infants [66.7%]; RR, 1.3 [95% CI, 1.1 to 1.5]), with no difference at 16 weeks (85 infants [88.5%] vs 80 infants [81.6%]; RR, 1.1 [95% CI, 1.0 to 1.2]). At 8 weeks, 19 RP mothers (20.4%) vs 9 control mothers (9.7%) usually or always put their infant to bed while still awake (RR, 2.1 [95% CI, 1.0 to 4.4]). At 16 weeks, RP infants were less likely than controls to be fed as the last activity before bed (18 infants [18.8%] vs 37 infants [37.8%]; RR, 0.5 [95% CI, 0.3 to 0.8]). RP infants were also more likely than controls to fall asleep to white noise at 8 weeks (21 infants [22.6%] vs 8 infants [8.5%]; RR, 2.7 [95% CI, 1.2 to 5.7]) and 16 weeks (17 infants [17.7%] vs 5 infants [5.1%]; RR, 3.5 [95% CI, 1.3 to 9.0]). RP mothers were more likely than controls to use dream feeds (ie, waking the infant to feed before mother goes to bed) at both 8 weeks (66 mothers [72.5%] vs 37 mothers [39.8%]; RR, 1.8 [95% CI, 1.4 to 2.4]) and 16 weeks (60 infants [62.5%] vs 42 infants [43.3%]; RR, 1.4 [95% CI, 1.1-1.9]). There were no group differences in the percentages of infants who fell asleep swaddled, with a pacifier, or while being held (Table 2). At 16 weeks, RP mothers were more likely than controls to report that their infant never sleeps in the same bed as an adult or another child (41 mothers [42.7%] vs 27 mothers [27.8%]; RR, 1.5 [95% CI, 1.0 to 2.3]). Other sleep safety practices did not significantly differ between groups.

### Night Waking and Feeding

At 8 weeks, there were no significant differences in the number of nighttime wakings or feedings (Table 3). By 16 weeks, infants from the RP group woke fewer times per night (mean difference, −0.4 [95% CI, −0.6 to −0.1] times per night) and were fed after waking fewer times during the night than control infants (mean difference, −0.3 [95% CI, −0.6 to −0.1] times per night). There were also some significant intervention effects on maternal responses to nighttime wakings. At 8 weeks, mothers in the RP group were less likely than those in the control group to play with their baby until they were ready to go back to sleep (8 mothers [8.6%] vs 18 mothers [19.2%]; RR, 0.4 [95% CI, 0.2 to 1.0]), watch television or videos with the baby until the baby fell asleep (5 mothers [5.4%] vs 16 mothers [17.0%]; RR, 0.3 [95% CI, 0.1 to 0.8]), or sing to the baby (8 mothers [8.6%] vs 18 mothers [19.1%]; RR, 0.4 [95% CI, 0.2 to 1.0]). At 16 weeks, RP mothers were more likely than controls to put their baby back down while awake after feeding (21 mothers [21.9%] vs 10 mothers [10.2%]; RR, 2.1 [95% CI, 1.1 to 4.3]) or give the baby a few minutes to fall back asleep on their own (33 mothers [34.4%] vs 21 mothers [21.4%]; RR, 1.6 [95% CI, 1.0 to 2.6]). There were no differences at either time point in the percentage of mothers who reported holding or rocking their baby back to sleep, rubbing or patting their baby without picking them up, giving a pacifier, or feeding their baby back to sleep (Table 3).

**Table 3.** Night Waking and Parenting Strategies

Measure	8 wk			16 wk			Model *P* value^a^	
	No. (%)		Estimate (95% CI)^b^	No. (%)		Estimate (95% CI)^b^	Group	Group × time
	RP (n = 93)	Control (n = 94)		RP (n = 96)	Control (n = 98)			
Night wakings, mean (SD), times per night	2.2 (0.8)	2.3 (1.0)	−0.1 (−0.4 to 0.1)^c^	1.5 (0.9)	1.8 (1.0)	−0.4 (−0.6 to −0.1)^c^	.07	.15
Night feedings, mean (SD), times per night	2.1 (0.8)	2.0 (0.9)^d^	0.0 (−0.2 to 0.3)^c^	1.6 (0.9)^e^	1.9 (0.9)^f^	−0.3 (−0.6 to −0.1)^c^	.22	.007
Response to infant night wakings								
Give few min to fall back asleep	27 (29.0)	24 (25.5)	1.1 (0.7 to 1.8)^g^	33 (34.4)	21 (21.4)	1.6 (1.0 to 2.6)^g^	.09	.14
Pick up and hold or rock back to sleep	48 (51.6)	43 (45.7)	1.1 (0.8 to 1.5)^g^	24 (25.0)	35 (35.7)	0.7 (0.4 to 1.1)^g^	.46	.05
Rub or pat but do not pick up	15 (16.1)	14 (14.9)	1.1 (0.6 to 2.1)^g^	22 (22.9)	15 (15.3)	1.5 (0.8 to 2.7)^g^	.28	.38
Feed back to sleep	47 (50.5)	44 (46.8)	1.1 (0.8 to 1.4)^g^	34 (35.4)	40 (40.8)	0.9 (0.6 to 1.2)^g^	.88	.28
Feed but put back down awake	22 (23.7)	18 (19.2)	1.2 (0.7 to 2.1)^g^	21 (21.9)	10 (10.2)	2.1 (1.1 to 4.3)^g^	.03	.18
Give pacifier	36 (38.7)	32 (34.0)	1.1 (0.8 to 1.7)^g^	35 (36.5)	31 (31.6)	1.2 (0.8 to 1.7)^g^	.43	.91
Bring to parent bed	3 (3.2)	3 (3.2)	1.0 (0.2 to 4.9)^g^	3 (3.1)	3 (3.1)	1.0 (0.2 to 4.9)^g^	.99	.98
Play with baby until ready to go back to sleep	8 (8.6)	18 (19.2)	0.4 (0.2 to 1.0)^g^	7 (7.3)	15 (15.3)	0.5 (0.2 to 1.1)^g^	.02	.90
Watch TV or video with baby until falls asleep	5 (5.4)	16 (17.0)	0.3 (0.1 to 0.8)^g^	9 (9.4)	18 (18.4)	0.5 (0.2 to 1.1)^g^	.005	.41
Sing to baby	8 (8.6)	18 (19.2)	0.4 (0.2 to 1.0)^g^	10 (10.4)	11 (11.2)	0.9 (0.4 to 2.1)^g^	.19	.10

Abbreviation: RP, responsive parenting.

^a^ *P* values are from generalized linear models, adjusting for infant weight-for-age *z*-score. These models exclude 11 participants for whom weight was not collected at 16 weeks due to COVID-19 pandemic restrictions.

^b^ Unadjusted, includes all available data.

^c^ Expressed as mean difference.

^d^ Among 93 participants.

^e^ Among 94 participants.

^f^ Among 89 participants.

^g^ Expressed as risk ratio.

## Discussion

This secondary analysis of the Sleep SAAF RP randomized clinical trial among Black families found that the intervention significantly improved several aspects of infant sleep and sleep-related parenting practices over the first 16 weeks post partum. Most notably, at 16 weeks, infants in the RP condition were sleeping 41 minutes longer at night and 73 minutes longer over the entire day, resulting in greater likelihood of meeting the minimum recommended sleep duration of 12 hours per day. Infants in the RP group were also waking less frequently during the night and were fed fewer times after waking by 16 weeks. RP mothers reported engaging more in a number of targeted sleep parenting practices relative to mothers assigned to the child safety control condition. Although results were not entirely consistent across the 8- and 16-week assessments, some significant differences were observed for earlier bedtimes, using a bedtime routine, putting baby to bed while awake, using white noise, and not feeding as the last activity before bed. Mothers also reported engaging in more developmentally appropriate responses to nighttime wakings, including putting baby back down while awake and waiting a few minutes to see if the baby fell asleep on their own, and engaged in less stimulating responses, such as playing with baby, watching TV, or singing. Importantly, these benefits were realized in the RP group without reducing safe sleep practices and achieved in the context of a relatively brief multicomponent intervention (2 sessions totaling approximately 2-3 hours). Overall, consistent with the INSIGHT study on which the Sleep SAAF RP intervention was based,^26^ these results provide evidence that the RP intervention had positive effects on infant sleep and enhanced many, but not all, responsive sleep parenting practices.

The positive effects of the RP intervention are particularly noteworthy in light of well-documented disparities in sleep experienced by Black individuals in the US throughout the lifespan^1,2,3^ and calls to reduce these disparities.^19,20^ There has been limited attention to interventions to promote better sleep for Black infants, as most early sleep interventions for this population have focused on SIDS prevention.^35,36,37^ To our knowledge, Sleep SAAF is the first study to show any benefits of a RP intervention on sleep and sleep practices among Black infants and their families. At the same time, we note there is still room for improvement: 35% of infants in the RP condition were not meeting sleep recommendations of at least 12 hours per day by age 16 weeks, and there were many domains in which RP mothers were no more likely than controls to implement recommended sleep guidance. Further understanding of barriers to implementation of intervention messaging and findings ways to overcome these challenges, as well as greater attention to socioecological factors affecting pediatric sleep disparities (eg, poverty, neighborhood characteristics),^19^ may further enhance infant sleep among this population.

### Limitations

This study has some limitations. First, all outcomes were reported by mothers using the BISQ, raising the potential for social desirability bias. Sleep duration and nighttime wakings on the BISQ are associated with actigraphy measures,^30^ but more objective measures would have strengthened our findings. Second, our sample was limited to primiparous Black mothers recruited from a single hospital in the southeastern US, calling for further research on other samples of Black families. Third, participants in the control condition also received an intervention that discussed infant sleep practices. That intervention was not intended to affect infant sleep duration or parenting practices, such as bedtime routines or responses to nighttime wakings, but this intervention may have influenced some outcomes (eg, percentage of babies put to sleep on their back or with a pacifier) relative to a no-treatment control condition.

## Conclusions

This secondary analysis of a randomized clinical trial found that the Sleep SAAF RP intervention for first-time Black mothers increased infants’ nighttime and total sleep duration, reduced nighttime wakings and feedings, and enhanced some aspects of mothers’ responsive sleep practices (eg, bedtime routines and behaviors). These findings provide initial evidence that RP interventions can enhance infant sleep among Black families and call for further research on whether these interventions can reduce early sleep disparities and promote better health for Black individuals in the US.

## Notes

Supplement 1.
Trial Protocol and Statistical Analysis Plan

Supplement 2.
eMethods.
**eTable.** Sleep SAAF 3 week sleep variables by study group
eReferences.

Supplement 3.
Data Sharing Statement
