# Supplementary material for: Effects of a Responsive Parenting Intervention Among Black Families on Infant Sleep: A Secondary Analysis of the Sleep SAAF Randomized Clinical Trial
Source: JAMA Netw Open. 2023 Mar 31;6(3):e236276. doi: 10.1001/jamanetworkopen.2023.6276 (PMC10066466; doi:10.1001/jamanetworkopen.2023.6276)
Supplement: Supplement 1. — Trial Protocol and Statistical Analysis Plan [file jamanetwopen-e236276-s001.pdf]

Protocol Title: Sleep-Safe: A Strong African American Families Study

Principal Investigator: Brian Stansfield, MD

1. Objectives

Describe the purpose, specific aims, and hypothesis:
Rapid weight gain in infancy is a risk factor for obesity and comorbidities later in the lifespan. This project is designed to test whether teaching mothers prompt, contingent, developmentally appropriate responsive parenting skills to soothe infants and promote adequate sleep can reduce rapid weight gain among African American infants living in the rural South. **Specific Aim 1: To assess the effects of responsive parenting, focused on infant sleep and soothing, on infant weight and reducing rapid weight gain from 2 weeks to 16 weeks among African American infants.** We hypothesize that infants in the sleep/soothe condition will have lower weight outcomes (i.e., BMI z scores, weight-for-length z scores, weight-for-age z scores) at age 16 weeks and gain weight more slowly from 2 to 16 weeks compared to infants in a safety control condition. . We consider whether weight-related factors (e.g., maternal pre-pregnancy BMI, gestational weight gain, intent to breast or formula feed, maternal smoking) moderate intervention effects. **Specific Aim 2: To assess effects of responsive parenting on parental and infant behaviors, and whether these mediate effects on infant growth.** We hypothesize that compared with control parents, parents in the sleep/soothe condition will show increases in responsive parenting, parenting self-efficacy, and the use of alternatives to feeding to soothe (white noise, pacifier, etc.), and decreases in feeding to soothe. Over time, infants in this condition will have longer sleep bouts and fewer feedings. These outcomes will be linked to healthier patterns of weight gain from 2 weeks to age 16 weeks, fully accounting for group differences. **Specific Aim 3: To examine moderation of intervention effects by individual and contextual factors.** We examine whether individual and contextual factors common among African American mothers moderate the effectiveness of the sleep/soothe intervention, informing development of future multi-component programs.

2. Background

Describe the background and rationale for the study:

This project is in response to the call for research to understand factors in infancy and early childhood that affect obesity development. Racial disparities in overweight among African American children compared to White children begin early in development and continue throughout the lifespan. During infancy, African American children have elevated rates of rapid weight gain and greater prevalence of high weight for length. Rapid weight gain is one of the most consistent risk factors associated with later overweight and obesity, showing associations with later overweight and obesity among toddlers, children, and young adults. Despite these well-established associations and the clear need for early intervention, there has been limited attention to developing interventions to prevent rapid weight gain among African American infants within underserved, low SES contexts. Accordingly, we propose to test whether teaching mothers prompt, contingent, developmentally appropriate responsive parenting skills to soothe infants and promote adequate sleep can reduce rapid weight gain among African American infants living in the rural South. Our examination of sleep and soothing evaluates a component of early obesity prevention programs that is promising, attractive to potential participants, and that may be particularly potent for African American infants in underserved, low SES contexts, making it the natural starting point for efforts to apply responsive parenting interventions in this high-risk context.

Findings from our two previous RCTs have provided evidence for efficacy of a multi- component responsive parenting (RP) program among middle income, predominantly White mother-infant dyads. PI Birch and colleagues tested whether a multi-component RP program, delivered using home visits and including guidance on infant sleeping, soothing, active social play, and feeding, including the transition to table foods during the first year of life, could reduce rapid weight gain during infancy and overweight in early childhood. Relative to a home safety control, infants randomized to the RP program demonstrated significant differences in sleep behaviors at 8-, 16-, and 40-weeks, including longer nighttime sleep duration, had more consistent bedtime routines and earlier bedtimes, and were more likely to self-soothe to sleep without being fed. Relative to control, infants of parents receiving the RP program gained weight more slowly in the first months after birth, had lower weight-for-length percentiles at 1 year, and were less likely to be overweight at 1 year.

Shorter sleep duration is more prevalent among African Americans and is related to multiple adverse health outcomes including obesity. In infancy, sleep duration and feeding are tightly linked; sleep bouts tend to be interrupted by feeding; higher feeding frequency is associated with shorter sleep duration. The responsive parenting sleep/soothe intervention has the potential to alter parenting and infant behaviors (sleep, soothing, and feeding), directly affecting rapid weight gain and ultimately long-term weight outcomes, by promoting appropriate, prompt, and contingent soothing responses to infant crying. Focus groups with African American mothers suggest that the intervention will be well received, but the potential for moderation of effectiveness by individual (depressive symptoms) and contextual (poverty, relationship support) stressors common to African American mothers is currently unknown. Examining these factors as they relate to maternal response and rapid weight gain among infants will increase our understanding of how stress affects high-risk samples, potentially identifying additional intervention foci to be targeted in a subsequent multi- component program optimized for this high-risk population. Likewise, testing the mediational model that has guided this research to date and determining its applicability in this sociocultural context is an essential step in optimizing future interventions.

Our conceptual model is shown below. As shown in the Figure, we argue that a responsive parenting sleep/soothe intervention (Sleep SAAF) has the potential to alter parenting behaviors (responsive parenting, parenting self-efficacy, alternatives to feeding to soothe) and infant behaviors (sleep, soothing, and feeding). These in turn affect rapid weight gain and ultimately long-term weight outcomes. However, the effectiveness of this intervention may be affected by individual and contextual stressors common to African American mothers, including depressive symptoms, socioeconomic and race-based stressors, and a challenging interpersonal context.

Figure 1. Conceptual Model: Early Factors Affecting Rapid Weight Gain among African American Infants

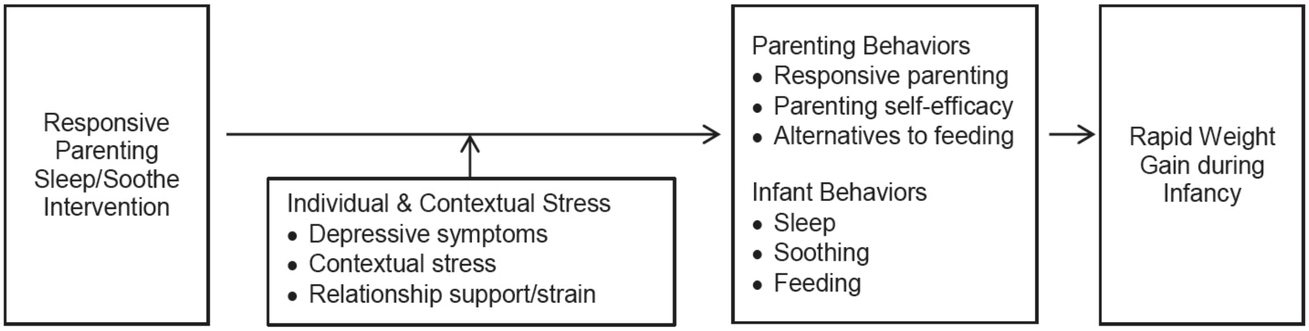

3. Inclusion and Exclusion Criteria

List the inclusion/exclusion criteria:

A total of 300 African American mother-infant dyads will be recruited from AU's newborn nursery. Co-participation of the mother-infant dyad is a study requirement. Paternal involvement is voluntary, but not necessary. If fathers are interested in participating, they will sign separate consents.

INCLUSION CRITERIA

Eligible mother-infant dyads for this trial will meet the following criteria:
1) full-term infant (≥ 36 0/7 weeks gestational age), apparently healthy and without significant morbidity
2) singleton infant
3) nursery/NICU/maternity stay of 7 days or less
4) Medicaid eligible

C. EXCLUSION CRITERIA

(not explicitly obvious from the inclusion criteria)
1) non-English speaking
2) infant birth weight <2500 grams
3) presence of a congenital anomaly or neonatal physical or metabolic condition that significantly affects a newborn's feeding (e.g. cleft lip, cleft palate, metabolic disease)
4) any major maternal morbidities, pre-existing condition that would affect postpartum care or her ability to care for her newborn (e.g., narcotic drug use: heroin, cocaine, meth, pain pills, etc; on chemotherapy; uncontrolled MS; uncontrolled depression causing social service contact).
5) plan for newborn to be adopted
6) plan to move from area within four months of delivery
7) residence further than 75 miles from Augusta, GA

4. Number of Subjects/Records/Samples Collected

Indicate the total number of subjects to be accrued/records reviewed/samples collected across all sites:

300 mother-infant dyads will participate in the study. All mothers will complete a series of questionnaires at enrollment and at 5 time points (study procedures described below). AU medical records will be used to gather selected demographic information on mothers (race, maternal age, gestational age, weight at delivery) and infants (date of birth, weight and length at delivery).

The study will be conducted over 5 years. The table below details the number of target participants per year and the number of research interactions each year (300 mother-infant dyads with 5 visits each):

Year	Participants	Research Interactions
Year 1	50	250
Year 2	100	500
Year 3	100	500
Year 4	50	250
Year 5	0	0
**Total**	**300**	**1500**

5. Recruitment Methods

Describe when, where, and how potential subjects will be recruited:

Recruitment of 300 African American mothers and their infants will take place at Augusta University Medical Center in the Children's Hospital of Georgia's mother/baby nursery, when potential subjects are newly postpartum. A dedicated recruitment coordinator will be hired (AU staff) and trained to identify (via active medical chart data), screen, and enroll qualified subjects.

The coordinator will have access to electronic medical records systems and will approach patients who match basic inclusion criteria. The recruitment coordinator will deliver an approved verbal script to determine mother's interest in participation. With the mother's agreement, the coordinator will complete approved screening protocol to determine eligibility. Records will be kept to track those who do not qualify as well as those who decline to participate. Qualified and interested participants will move through the consent and enrollment process with the recruitment coordinator. j

6. Procedures Involved

a. *Describe the procedures involved to include those procedures that are standard evaluation and/or care and those that are solely for research purposes:*

All procedures are solely for research purposes.

Enrollment at AU Hospital Newborn Nursery (after delivery, Recruitment Coordinator):

1. Obtain maternal informed consent for mother-baby dyad

2. Obtain paternal informed consent for his own participation (height and weight measurement, romantic relationship questionnaires, coparent questionnaires, depressive symptoms)
3. Obtain selected demographic information (race, maternal age, gestational age, weight at delivery and (self-reported) pre-pregnancy weight) from the AU medical record system
4. Complete enrollment data collection forms
    a. Chart abstraction form (coordinator completed)
    b. Demographics and Health History Questionnaire (coordinator interview with mother)
    c. Contact form
5. Measure weight (kg) and height (cm) of mothers and fathers (if father not present, will attempt to measure at subsequent visit)
6. Measure infant anthropometry: length (cm), weight (g), head circumference (cm).
7. Schedule the first research visit at the home for 1 week postpartum

<u>Research Visit 1: (Home, 7-10 days postpartum, Community Research Associate (CRA))</u>

1. Administer initial infant sleep safety information and materials
2. Randomize mother-infant dyads to either the control or the intervention group
3. Complete home environment assessment
4. Complete questionnaire measures
5. Measure infant weight and length
6. Measure maternal weight
7. Measure paternal weight

<u>Intervention Visit 1: (Home, 2 weeks, CRA)</u>

1. Deliver intervention or control group program
2. Complete questionnaire measures
3. Measure infant weight and length
4. Measure maternal weight
5. Measure paternal weight

<u>Intervention Visit 2: (Home, 8 weeks, CRA)</u>

1. Deliver intervention or control group program
2. Complete questionnaire measures
3. Measure infant weight and length
4. Measure maternal weight
5. Measure paternal weight

<u>Intervention/Research Visit: (Home, 12 weeks, CRA)</u>

1. Deliver intervention or control group program
2. Provide mother with activity monitor
3. Complete questionnaire measures
4. Measure infant weight and length
5. Measure maternal weight
6. Measure paternal weight

<u>Research Visit 3: (Home,16 weeks, CRA)</u>

1. Complete questionnaire measures
2. Collect activity monitor from mother
3. Measure infant weight and length
4. Measure maternal weight
5. Measure paternal weight

*** Table 1 provides additional details on scheduled assessments at each time point. We provide additional information on the intervention and control group materials in the following section on study design. Refer to Appendix 4 – Measures***

Table 1. Evaluation Schedule of Assessments and Measures Used

	Infant Age and Visit Description				
	1 Wk	2 Wks	8 Wks	12 Wks	16 Wks
**Infant Growth**					
Weight & length	X	X	X	X	X
**Infant Sleep, Soothe, and Feeding Outcomes**					
Infant sleep	X	X	X	X	X
Infant temperament					X
Feeding frequency	X	X	X	X	X
**Parenting Outcomes**					
Parenting self-efficacy				X	X
Soothing practices				X	X
Maternal feeding practices				X	X
Family routines				X	X
**Child Safety Outcomes**					
Safe sleep practices	X				X
Child safety practices	X				X
**Stress-Support Moderators**					
Maternal depressive symptoms	X				X
Socioeconomic stressors	X				
Race-based stressors	X				
Romantic relationship characteristics	X				X
Coparent relationship characteristics	X				X
Social support	X				
**Demographics and Health**					
Family demographics and maternal/infant health	X				
**Intervention-Related Variables** [a]					
Coparent involvement in intervention		X	X	X	
Implementation quality		X	X	X	

<u>Infant Growth</u>

Infant weight and length. At each home visit, infant weight and recumbent length/height will be measured by research staff trained in obtaining anthropometrics, and will be used to calculate weight-for-length and BMI percentile and BMI Z-score based on percentiles for age and sex established by the WHO (for outcomes <2 years) and CDC (for outcomes 2 years and up). Weights will be obtained using Seca Model 334 and 876 scales. Recumbent lengths will be obtained in triplicate with Seca Model 232 recumbent length board and Seca 240 mechanical, high precision measuring rod.

<u>Infant Sleep, Soothe, and Feeding Outcomes</u>

Infant sleep. The validated Brief Infant Sleep Questionnaire (BISQ) will be used to assess sleep. This survey assesses infant sleep location, before-bedtime activities, and sleep patterns. Sleep duration is divided into nighttime (7 pm–7 am) and daytime (7 am–7 pm) and is reported in hours and minutes. Selected questions capturing sleep duration (day and night), location, and night feedings will also be included. We also plan to use actigraphy data from mothers to capture their awakening as a complement to maternal self-report.

Infant temperament. Infant temperament will be assessed using the 10-item Pictorial Assessment of Temperament. This measure demonstrates strong convergent and predictive validity and has previously been used in 7-week old infants. Infant difficultness, characterized by fussiness and difficulty soothing, has been related to rapid weight gain, body composition, and increased obesity risk.

Feeding. The "Babies Need Feeding" scale from the Baby's Basic Needs Questionnaire was used in our previous studies to examine current feeding mode (breast milk and/or formula), percent of daily feedings that are breast milk or formula, as well as the use of bottle feeding of formula or human milk. We will also include the widely-used CDC Infant Feeding Practices Survey to allow for a more detailed examination of feeding.

<u>Parenting Outcomes</u>

Parenting self-efficacy. The validated 15-item Karitane Parenting Confidence Scale measures perceived parental self-efficacy in the parents of infants aged 0-12 months. This measure will allow us to test whether parents' feelings of self-efficacy mediate intervention effects and whether parenting self-efficacy relates to the behavioral variables of interest.

Soothing practices. The "Babies Need Soothing" scale from the Baby's Basic Needs Questionnaire measures the extent to which parents use feeding for reasons other than in response to hunger, such as to soothe, calm, or control behavior. This measure assesses: (1) reasons for crying, (2) frequency and effectiveness of soothing techniques, including feeding, (3) foods used to soothe, and (4) contexts where food to soothe is used.

Maternal feeding beliefs and practices. The Infant Feeding Styles Questionnaire is a validated self-report instrument that assesses maternal feeding beliefs and behaviors. It was developed specifically among low-income African American mothers. The Baby Eating Behavior Questionnaire (BEBQ) will be used to measure maternal perception of infant hunger and satiety.

Family routines. We will assess the extent to which parents report that there is order and routine versus chaos and confusion in their home using selected questions from the Confusion, Hubbub, and Order Scale.

<u>Child Safety Outcomes</u>

Safe sleep practices. We will assess safe sleep practices using a 6-item measure evaluating the frequency of sleep practices such as how often the baby sleeps with toys, with an adult, and on her back.

Child safety practices. The Framingham Safety Survey for the first year of life will be administered to assess high-risk behaviors or conditions reported by parents that occur in the home. It will be used to evaluate the impact of the Child Safety Control. This survey was adopted by the AAP and is a screening tool for injury prevention.

Stress-Support Moderators

Maternal depression. We will assess maternal depressive symptoms using two scales: the Edinburgh Postnatal Depression Scale, a measure of depressive symptoms valid for postnatal women, as well as the widely used Center for Epidemiological Studies Depressive Scale.

Maternal trauma history. We will assess mothers' history of childhood trauma with the widely used Childhood Trauma Questionnaire – Short Form (Bernstein et al., 2003)

Socioeconomic stressors. Socioeconomic stressors will include a range of socioeconomic measures assessing individual and community level factors. Individual characteristics will include: (1) Socioeconomic Status, which combines education and income; and (2) Financial/Employment Situation, which will include multiple widely used scales that focus on financial adjustments, financial hardship, unmet needs, and employment. Community characteristics will include (1) Community Disadvantage, which will include the sum of 5 census variables used in previous research: % below poverty line, % single-parent families, % public assistance, % < high school education, and median family income; and (2) Social Isolation, which will include the combination of 3 census variables: % below poverty line, % living in the same house over years, and % housing occupied by owners.

Race-based stressors. We will include four measures of race-based stressors: (1) Discrimination, using the18-item long form of the widely used Schedule of Racist Events, which assesses both personal and vicariously-experienced discrimination, (2) Perceived Racism, using the 15-item index based on Sellers et al. (1997) Public Regard scale, (3) Internalized Racism, using the 24-item Racist dimension of Taylor & Grundy's (1996) Scale, and (4) Racial Segregation, using the % of racial groups in census tracts based on Massey and Denton's (1988) formula.

Romantic relationship characteristics. All mothers will report on their current romantic relationship status, including (a) married and cohabiting, (b) married but not cohabiting, (c) cohabiting, (d) romantically involved on a steady basis but not living together, (e) involved in an on-again and off-again relationship, and (f) single. Categories (d) and (e) were used in the Fragile Families and Child Wellbeing study to capture greater variability in the relationships of low-income families. Mothers who indicate that they are involved in a current romantic relationship will be asked whether this relationship is with the child's father or with another romantic partner.

Mothers who are involved in a romantic relationship will be asked to rate the following: (1) Relationship Satisfaction, using the 4-item Couples Satisfaction Index, (2) Conflict with Romantic Partner, using the 5- item Hostility Scale, (3) Warm, Caring, and Affectionate Behaviors, (4) Dedication Commitment, a 4-item scale assessing the desire to persist in the relationship despite obstacles, (5) Dissolution Risk, two items assessing thoughts of ending the relationship, and (6) Relationship Length and History, including how many times the current relationship has ended/resumed. If fathers are available and interested in participating, they will also complete these questionnaires.

Coparent relationship characteristics. All mothers will also report on who is involved in coparenting (e.g., child's father, child's grandmother). Our feeding surveys will assess the proportion of feedings given by the mother as well as other caregivers. We will also assess aspects of the coparenting relationship (e.g., "My partner and I have different ideas regarding our child's eating, sleeping, and other routines") using selected questions from the Coparenting Relationship Scale. If fathers are available and interested in participating, they will also complete these questions.

Social support. Mothers will also be asked to rate the social support they receive from people other than their partner and/or coparent using the Social Provisions Scale.

Demographics and Health

Family demographics and maternal/infant health. Parent and demographic covariates will include: Pre- pregnancy BMI, smoking during pregnancy, gestational weight gain, maternal type 2 or gestational diabetes mellitus, maternal and paternal BMI assessed at the first research visit, income, education, and employment (work status, hours worked). Infant covariates will include: Infant weight at age 1 week (the first assessment) adjusted for gestational age, child sex, and feeding mode (predominantly breastfed if 80% or more of milk feedings were breastmilk, predominantly formula-fed if 80% or more of milk feedings with formula milk, or mixed-feedings).

Parent weight and height. Mothers' pre-pregnancy weight and weight prior to delivery will be obtained from chart abstraction, if available. Should these data be missing from charts, it will be collected via survey. Mother's height will be measured using stadiometer at AU and weight will be measured using a calibrated scale at AU. At all subsequent visits, mothers and fathers will again be weighed using calibrated scales.

Intervention-Related Variables

Coparent involvement in intervention. While paternal and/or coparent involvement in the study is not required for participation, fathers and/or coparents will be strongly encouraged to attend study visits so they too can receive the study intervention. We will collect data on their attendance and degree of participation in the intervention visits.

Implementation quality. Our previous evaluations gave us the opportunity to evaluate participants' responses to training and evaluation materials and this has led to revised and simplified training and assessment tools, suitable for those reading at a 6th grade level. As in our previous studies, we also include measures immediately after study visits to systematically assess the quality of intervention staff's implementation of the intervention. These measures will be completed by the Community Research Associates (CRAs) delivering the intervention (see below). These data will provide information on overall implementation quality in our sample and will also allow implementation quality to be investigated as moderator of intervention effects. We will use parallel measures with the child safety control group to assess implementation quality.

b. *Describe and explain the study design:*

The study design is a randomized controlled trial. This trial will deliver a responsive parenting intervention (Sleep SAAF) that builds upon our previously tested Soothe/Sleep curriculum to provide information on safe sleep practices, how to soothe, how to distinguish hunger from other distress, how to promote self-soothing, and bedtime routines. The responsive parenting intervention will be compared to a safety control group. To promote uptake of program content, the programs will be delivered across 3 sessions at **2-weeks, 8-weeks, and 12-weeks postpartum**, allowing for increased dosage of this critical component. Assessments will be conducted at 1-week postpartum, 2 weeks postpartum, 8 weeks postpartum, 12 weeks postpartum, and 16-weeks postpartum (final growth outcomes).

The intervention group will be compared to a child safety control group receiving an equal number of visits, which will be focused on child and sleep safety and should not affect infant weight gain. Both interventions will be delivered in the home setting by African American community research associates (CRAs) who are employed and trained by UGA's Center for Family Research (CFR). CRAs will also

conduct data collection visits at participants' homes; when possible, the research visits (1 and 16 weeks) and the intervention visits (2, 8 and 12 weeks) will be conducted by different CRAs. Self-report data will be gathered via audio computer-assisted self-interviewing (ACASI) software on laptop computers. ACASI elicits less social desirability bias and more accurate reports on sensitive issues than face-to-face interviews or written surveys. Video and audio enhancements guide respondents through the survey; literacy is not an issue. CRAs will receive 16 hours of training on informed consent, building rapport, and helping participants to use the survey. They will meet regularly with the Project Coordinator and participate in yearly refresher trainings. Our experienced data collection staff have successfully implemented these protocols in multiple projects.

All participants will receive information on proper infant sleep hygiene and creating a safe sleep environment, with a focus on "back to sleep," supine placement of the infant for sleep. We will use materials from NICHD's "Babies Sleep Safest on Their Backs: A Resource Kit to Reduce the Risk for SIDS in African American Communities", which was developed based on guidelines from the American Academy of Pediatrics. Guidelines include always placing baby on her back, placing on a firm sleep surface, not smoking around baby, and keeping baby's sleep area close to, but separate from, where mother and others sleep. For all participants, the sleep surface will be inspected and parents will be provided a bassinet if they do not already have one.

In addition to this information on safe sleep practices, participants in the intervention condition will receive guidance on responsive parenting, and the control group will receive additional content on child safety. Additional details are provided below.

Responsive Parenting Group. The Sleep SAAF responsive parenting intervention includes setting appropriate expectations regarding normal infant development and need for care during the first months of life, including infant sleep-waking, active alert behavior, crying, and feeding. Guidance on caregiving focuses on (1) how much sleep infants need and how to help them get it, (2) avoiding the use of feeding as the default response to infant crying, (3) how to discriminate hunger from other reasons for infant crying (too warm or too cold, too tired, diaper change needed), and (4) how to use alternative soothing strategies [including use of white noise, movement, side/stomach positioning while being held, non-nutritive sucking (pacifier), swaddling] to discriminate among causes of crying and to soothe a crying infant, as well as how to cope with crying. We will provide information about normal sleep patterns in infants. Guidance on how to help infants get the sleep they need will include establishing a bedtime routine that includes putting the infant to bed early, putting baby to bed drowsy but awake, dealing with night waking to promote self-soothing, and using "5Ss" to calm the baby. We will also discuss avoiding feeding the infant to sleep or putting the infant to bed with a bottle.

The Sleep/Soothe curriculum also includes some basic information on feeding, including normative information on how much and how often young infants typically eat, and on how to discriminate infant hunger from other distress and to recognize when their baby is full. Because many caregivers assume that a fussy infant is hungry, feeding is often their first response to fussing and crying. During the intervention visits, intervention staff will work with parents of breastfeeding and formula feeding infants, as well as those feeding pumped breast milk, to recognize hunger cues (rooting, mouthing, bringing hand to mouth) and fullness cues (letting go of nipple, falling asleep, turning head away, interest in other things). Excerpts from an instructional video will be shown to parents to illustrate infant behaviors indicative of hunger and fullness. Expectations for typical feeding frequency during the day and night for breastfed and formula fed infants will be discussed. Parents will be given education on bottle sizes, milk/formula volumes, use of slow flow bottle nipples for infant under 4 months to prevent overfeeding or choking, and how to use fullness cues, rather than the amount of milk in the bottle, to determine when to terminate a feeding. Instructions will also advise parents to

delay introduction of other beverages until age 6 months and to avoid addition of infant cereal to a bottle unless explicitly instructed to by a physician.

Having emphasized hunger and satiety cues, intervention staff will then provide training to parents in how to use alternative soothing strategies for non-hunger-related infant crying, including how to swaddle an infant, use of a pacifier, white noise, and use of movement and positioning. Intervention staff will discuss several methods, which can be used separately or in combination. Parents will be provided with video clips from "The Happiest Baby on the Block," as was done in our previous studies. During home visits, parents will be coached in the use of these strategies with their infant.

Child Safety Control Group. For the child safety control group, in addition to guidance on safe sleep (described above), several aspects of newborn safety will be discussed, guided by information from The Injury Prevention Program (TIPP) from the American Academy of Pediatrics as well as the Academy's guide for health supervision, *Bright Futures*. Following TIPP guidelines for ages 0-4, information will include prevention of car injuries, falls, burns, choking, and suffocation. To prevent car injuries, child safety seat installation will be reviewed. To avoid falls, parents will be reminded to never leave a baby alone on a changing table, bed, sofa, or chair. To prevent burns, parents will be encouraged to never carry a baby and hold a hot liquid or food at the same time; home smoke detectors and water heater will be evaluated. The information covered by the safety intervention is typically presented as part of standard pediatric office care, but will be delivered in a more hands-on fashion at the home visits for the child safety control group.

Intervention materials include materials from the American Academy of Pediatrics, Centers for Disease Control and Prevention, and other sources as well as videos and duplication. Other expenses include bassinets to be distributed to all study participants. Mothers receiving the intervention will receive group appropriate tools such as swaddle blankets, white noise machine, pacifiers, etc. Mothers in the safety control group will receive group appropriate materials, e.g., first aid kits.

Participating mothers will be provided with a $75 incentive following the completion of each of two research visits (1 week, 16 weeks) as well as the combined research/intervention visit (12 weeks). A $25 incentive will be provided at the end of each intervention visit (2 weeks, 8 weeks). Total monetary incentive offered for each participant is $275. Each visit is expected to require 90-120 minutes and will include completion of project paperwork, documentation of informed consent, and the ACASI interview or the prescribed intervention activities. This incentive structure is in accordance with other IRB-approved participation incentives offered by CFR and is informed by feedback from community members.

c. *Describe the procedures performed to lessen the probability or magnitude of risks:*

As the current study is not blinded, the investigators will monitor for an increase in adverse events in each of the experimental conditions. Drs. Stansfield, Birch, and Lavner will assume all responsibility for addressing adverse events. Because the study will include children, all IRB requirements for the protection of children will be fulfilled.

All participants will receive a handout at the first visit with a list of community resources, including public health, mental health, food banks, shelters, division of labor, etc.

All information obtained by project staff about the project participants including survey data and disclosures or observations during the assessment visits will be held strictly confidential. However, in

the event of suspicion of child abuse, or concern about danger to self or others, project staff will be instructed to seek consultation from one of the PIs or the Project Coordinator. If one of these administrators agrees that the circumstances are of concern, an appropriate report will be made to either DCFS (in the case of suspected child abuse) or to 911 (in the case of danger to self or others).

Individual participant growth will be closely monitored by the investigators in order to identify growth failure. During each study visit, staff will calculate weight percentiles, and each child's growth chart will be plotted to allow for identification of potentially concerning growth patterns in real time. The infant's growth will be plotted on the CDC charts for this screen. Dr. Stansfield will review the growth chart within 1 week of the visit. There will be two initial screening criteria for growth concerns: (1) weight-for-age below the 5th percentile using growth charts from the WHO, and (2) downward crossing of a major percentile lines between any two study visits on the WHO weight-for-age growth chart statistically evaluated as a -0.67 Z-score change in order to provide a consistent measure across subjects. For any individual child who meets initial screening criteria for growth concerns, numerous factors will be considered in determining whether the child's growth is problematic and/or related to study condition. Examples of such factors include genetic potential based upon parental size, the participant's linear growth, feeding mode (breastmilk vs. formula), and interval illnesses. For predominantly breastfed infants, the WHO growth charts will be used to assess growth as these charts were designed specifically to monitor breastfed newborns and infants though the study's outcomes will be reported based upon percentiles on the CDC charts as is currently standard of care in the U.S.

The study team will report to the infant's primary care provider (PCP) if either of the two above screening criteria is met. The study's informed consent document will include information indicating that the study team will communicate with the participant's PCP; because all participants are infants, it is expected that they will all have a PCP or office where they receive their medical care as is typical for infants. Doing so will allow for open lines of communication between the study team and the PCPs in the event of concerns related to growth. If either the PCP or the study investigators believe that it is possible that these growth patterns are a negative result from study participation, the child will be withdrawn from the study. Additional details regarding growth monitoring are included in Section 8 (Provisions to Monitor the Data to Ensure the Safety of Subjects) below.

d. *Describe the duration of an individual subject's participation in the study and the time involved:*

Mother-infant dyads are enrolled for a period of 16 weeks. Interactions include initial enrollment and 5 subsequent visits to the family's home. Enrollment will take place within 24-72 hours of delivery (while the mother is admitted to the newborn nursery) and participation will continue until the infant is 16 weeks (not more than 19 weeks) of age. Initial enrollment procedures will take 60-90 minutes; each following study visit will take 90-120 minutes.

7. Data and Specimen Management

a. *Describe the data analysis plan, including any statistical procedures:*<br><br>Data analysis will be led by Dr. Lavner, who has expertise in the analysis of longitudinal data. Tianyi Yu, Ph.D., Assistant Research Scientist and Statistician at CFR, will provide statistical support for the conduct of analyses. All primary statistical analyses will invoke the intent-to-treat paradigm, analyzing data based on randomized assignment. Missing data will be handled using full-information maximum likelihood methods, which use all available information to estimate parameters, making this approach more efficient and less biased than other methods when data are missing at random. All models considered allow for the inclusion of relevant covariates. Covariates to be considered in refining the analyses, in addition to those described in detail in the proposed analyses below, include demographic factors (e.g., maternal employment and hours worked, primiparous/multiparous status, gestational diabetes), weight-related factors (e.g., maternal pre- pregnancy weight/BMI, gestational weight gain, maternal smoking), and intervention-related factors (e.g., implementation quality, coparent involvement in intervention). We will also examine (1) infant sex as a covariate, given different growth charts for boys and girls and (2) feeding mode as a covariate, given differences in weight gain between breastfed and formula-fed infants.<br><br>***Specific Aim 1: The Effects of Sleep SAAF on Infant Weight and Rapid Weight Gain from 2 Weeks to 16 Weeks***. In Specific Aim 1 the primary outcomes are infant weight and changes in weight. BMI is generally assumed to be the standard growth measure for assessing obesity-risk in children age 2 years and older, but there is not one universally accepted measure for children younger than age 2 years. Recent data suggests that infant BMI at age 2 months is more strongly associated with obesity at age 2 years than is weight-for- length. Accordingly, BMI z scores will be our primary outcome, but we will also consider models that examine a range of other weight outcomes, including weight for length z-scores, percentiles, and weight for age z-scores; we anticipate that change across different indices of weight gain will be highly correlated and lead to similar conclusions in tests of program impact as well as tests of potential moderators and the hypothesized mediational model.<br><br>First, we will examine differences in BMI and other weight outcomes at 16 weeks between the Sleep SAAF group and the control group using a linear mixed-effects model. The linear mixed-effects model will include intervention group and infant weight at age 1 week adjusted for gestational age. These analyses will test whether the Sleep SAAF group and the control group differ in their weight outcomes at age 16 weeks, which has previously been linked to child obesity risk at age 2 years.<br><br>Next, we will consider growth models that examine rate of change in weight from 2 weeks to 16 weeks. We will conduct growth curve analyses using hierarchical linear modeling to test whether the Sleep SAAF and control groups differ in their rate of change in weight over time, controlling for weight at 2 weeks. The model will include intervention group, time of growth measure, and infant weight at age 1 week adjusted for gestational age. These analyses will evaluate whether the Sleep SAAF group shows less of an increase in weight over time compared to the safety control group.<br><br>As an additional test of rapid weight gain, weight gain z-scores will be calculated for each measurement subsequent to the first intervention session at age 2 weeks (8 weeks, 12 weeks, and 16 weeks). Rapid weight gain scores will be calculated as an	☐ N/A

increase in weight-for-age z-score > +0.67, as described by Ong and Loos. This increase corresponds to crossing adjacent major centile lines on the standard CDC growth charts. The analysis for these rapid weight gain scores will be a repeated measures analysis, assessing the intervention effects on rapid weight gain scores from age 2 weeks up to age 16 weeks. The analysis will use generalized estimating equations (GEE) with a logit link, an extension of logistic regression which allows for repeated measurements on binary data, and will include infant weight at age 1 week adjusted for gestational age as a covariate. Because it is unlikely that within an 16-week period we will have many infants who meet the criteria for rapid weight gain based on the upward percentile crossing approach, we will also calculate conditional weight gain scores (CWG) following the method of Griffiths and colleagues, as described in our recent work.

Specific Aim 2: The Effects of Sleep SAAF on Parental and Infant Behaviors that Mediate Effects on Infant Growth. In Specific Aim 2 the primary outcomes are parental behaviors (parental self-efficacy, sleep/soothing practices, feeding practices, family chaos) and infant behaviors (sleep, soothing, and feeding) targeted by the intervention that may mediate effects on infant growth. We examine these effects in several steps.

First, we will examine differences between the Sleep SAAF and control groups in these outcomes at three time points post-intervention (8-, 12-, and 16- weeks postpartum) using the linear mixed-effects model described above. The linear mixed-effects model will include intervention group and infant weight at age 1 week adjusted for gestational age. These analyses will test whether the Sleep SAAF group and the control group differ in these behaviors post-intervention and will be used to identify variables for the formal test of mediation.

Second, we will examine whether parent and infant behaviors mediate infant weight outcomes. We will test these hypotheses using structural equation modeling (SEM). The first step in demonstrating mediation is to establish the effects on mediating and distal outcomes, as we described above. We then specify mediators as indirect effects in a path model. Intervention condition will be dummy coded and specified as a predictor of infant and parental behaviors, which in turn predict infant weight and weight gain. The significance of the mediating process will be tested using the Sobel or bootstrapping methods.

Specific Aim 3: Moderation of Intervention Effects by Individual and Contextual Factors. In Specific Aim 3 we consider whether individual (depressive symptoms) and contextual (socioeconomic stressors, race-based stressors, romantic relationship characteristics, coparent relationship characteristics, social support) factors at 1 week postpartum moderate program effects. These factors will be included in the analyses described above to determine whether they moderate intervention effects on weight outcomes (Specific Aim 1) and parental and infant behaviors (Specific Aim 2).

As an exploratory analysis, we will also evaluate whether the individual and relationship variables differ between the Sleep SAAF group and the control group at 16 weeks postpartum. Doing so will allow us to examine whether improving infant sleep and soothing has secondary benefits for mothers’ individual and relationship functioning.

b. *When applicable, provide a power analysis:*<br><br><u>Power</u><br>The primary outcomes for this study will be BMI z scores at 16 weeks (Specific Aim 1) and parent and infant behaviors at 8 weeks and 12 weeks (Specific Aim 2). We used G Power 3.1.9.2 to estimate the smallest intervention effect size we can detect with 80% power, a 5% two-tailed Type 1 error rate, and two groups of 150 each (conservatively assuming 10% attrition over the course of the study). We can achieve 80% power with an effect size *d* of 0.34. Our recent research showed an effect size of approximately 0.4 for infant nighttime sleep duration at 8 weeks and for infant conditional weight gain at 6 months, suggesting that we will have sufficient power in our sample to detect intervention effects.	☐ N/A
c. *Describe how data and specimens will be handled:*	☐ N/A
i. *What information will be included in that data or associated with the specimens?*<br><br>Subject data, including measurements, questionnaire data and intervention evaluation data will be maintained for analysis of study outcomes. The staff at AU and UGA will coordinate the creation of all paper and electronic data collection and management forms that will be distributed to research subjects and subsequently returned to researchers. Aside from consent/permission documents and contact information, all study data will be de-identified to ensure confidentiality. Upon enrollment, subjects will be assigned a study ID number which will serve as a code associated with all collected data. This code will be linked to the subject in only one document, the project directory.	
ii. *Where and how data and/or specimens will be stored?*<br><br>Electronic participant data will be stored on password protected computers, with all files also protected by password. Original and all copies of paper or hard copy data files will be stored on site (AU or UGA) in locked file cabinets. De-identified data will be stored separately from any files that may contain subject names. The project directory will be the only document containing a key code linking subjects to study ID numbers; this directory will be password protected. Only pertinent study team members (PIs, coordinators) will have access.	
iii. *How long will the data and/or specimens be stored?*<br><br>Participant data collected for this research will be analyzed for many years. Since it is not possible to know how long this analysis and follow-up will take, the research team will maintain access to these data indefinitely.	
iv. *Who will have access to the data or specimens?*<br><br>Only pertinent study team members (PIs, recruitment and project coordinators) will have access to the project directory. Other study team members (per IRB record), including graduate students, CFR team and statistical analysis team, will have access to raw data to be entered and analyzed per the study outcomes. These study team members will only have access to de-identified data.	

v. Who is responsible for receipt or transmission of the data and/or specimens?

Only pertinent and appropriate (as specified in section iv.) study team members will receive or transmit collected data for storage and analysis. Data will be entered via audio computer-assisted self-interviewing (ACASI) software on laptop computers or tablets, and will subsequently be transmitted to secure AU or UGA facilities as soon as practically possible by the Project Coordinator.

vi. How will data and/or specimens be transported?

As noted above, most data from the research visits will be collected using laptop computers or tablets, reducing the need for paper or hard copy data. If rare instances when this information is necessary, paper and hard copy data will be transported as needed by pertinent study team members. Data obtained via home visits will be delivered to secure AU or UGA facilities as soon as practically possible. Electronic data will be shared among study team members using a secure internet connection and an approved data management software program that will be password protected.

8. Provisions to Monitor the Data to Ensure the Safety of Subjects

This study involves no more than minimal risk study and this section is not required. ☐ **N/A**

The plan might include establishing a data monitoring committee and a plan for reporting data monitoring committee findings to the IRB and the sponsor.

a. Describe the plan to periodically evaluate the data collected regarding both harms and benefits to determine whether subjects remain safe.

The data and safety monitoring plan (DSMP) for this trial focuses on close monitoring of infant growth by the principal investigators (PIs) in conjunction with a Data and Safety Monitoring Board (DSMB), along with prompt reporting of excessive adverse events and any serious adverse events to the NIH/NIDDK and to the Institutional Review Board (IRB) at Augusta University (AU). Because behavioral interventions aimed at reducing rapid weight gain could theoretically result in insufficient weight gain by study participants, individual participant growth will be closely monitored by the investigators. The adverse event form, which we used in our previous studies, meets the goals of this plan.

In this study, an adverse event shall be defined as any detrimental change in the participant's condition, whether it is related to study interventions, study outcomes, or to another unrelated illness. Adverse effects may be (a) unrelated to the study interventions or (b) potentially related to the study interventions.

Adverse Events Unrelated to the Study Interventions:

Adverse events due to illnesses unrelated to study interventions may be grounds for withdrawal if the illness is considered significant by the study investigators or if the participant is no longer able to effectively participate in the study. A significant illness would be one that would compromise the child's ability to function normally and thrive, such as the diagnosis of a

malignancy, illness characterized by growth problems, disease requiring ongoing and intensive treatment, and/or one requiring repeated hospitalizations or physician visits. Subjects experiencing routine, minor, self-limited acute illnesses that typically occur during infancy and do not affect long-term growth will not be recorded, and the child will continue to participate in the study. Examples of minor illnesses include acute otitis media, bronchiolitis, upper respiratory infections, urinary tract infections, and gastroenteritis. Medications for acute, self-limited illnesses such as those stated above will not be recorded, but chronic medication use (> 1 month) will be recorded.

Other adverse events that could affect growth include a milk-protein allergy, other food allergies, or physician-diagnosed gastroesophogeal reflux disease (GERD) requiring medication. Surgical conditions such as intestinal malrotation and pyloric stenosis also would impact infant feeding and weight gain. Therefore, these will be recorded and if the participant's physician determines that these conditions can affect long-term growth, the participant will be withdrawn from the study. Documentation of an adverse event unrelated to study interventions that are not considered minor illnesses of childhood and those that can significantly affect growth will be recorded on an Adverse Event Report Form and will include the following information:

1. Description of the illness
2. Dates of illness
3. Treatment of illness and dates (medications, doses, and dose frequency)
4. Whether emergency treatment or hospitalization was required
5. Treatment outcome

Adverse Events Potentially Related to the Study Interventions:

It is theoretically possible that behavioral interventions designed to prevent rapid weight gain during infancy could result in underfeeding by parents and insufficient growth. The NIH-funded studies that serve as the foundation for the current project did **not** find an association between study interventions and these adverse events. While the results of these previous projects are reassuring, weight status and growth will be closely monitored and evaluated at frequent intervals in the proposed project in order to monitor the study intervention for potential adverse events. In the current study, there are several ways a potential adverse event related to growth will be identified:

1. Diagnosis by a treating primary care provider (PCP) of insufficient growth or failure to thrive
2. Weight-for-age below the 5th percentile using growth charts from the WHO
3. Downward crossing of two major percentile lines between any two study visits on the WHO weight-for-age growth chart statistically evaluated as a -0.67 Z-score change in order to provide a consistent measure across subjects

For the PCP diagnosis portion of identifying potential adverse events, the study's informed consent document will include information indicating that the study team will communicate with the participant's PCP. Because all participants are infants, it is expected that they will all have a PCP or office where they receive their medical care as is typical for infants. The study team will ensure lines of communication exist with PCPs in addition to the growth monitoring performed as part of the study. To establish the lines of communication between the study team and the PCPs, upon participant randomization, PCPs will be notified that their patients are participating in a study that includes an intervention arm designed to reduce rapid weight gain during the first 16 weeks after birth. A brief description of the study will be shared with them as well as a contact number to call the study team should the PCP become concerned about insufficient growth by their patient. Recognizing that growth monitoring is typically performed at all regularly scheduled primary care appointments, we will ask PCPs for their collaboration in monitoring infant growth in that we would like to be alerted to any concerns that their patient is demonstrating insufficient weight gain. In turn, the study team will report

to the PCP if we determine that their patient meets one of the two screening criteria above when assessed at study visits at 2 weeks, 8 weeks, 12 weeks, or 16 weeks.

During each study visit, home visit staff will calculate weight and length percentiles, and each child's growth chart will be plotted to allow for identification of potentially concerning growth patterns in real time. The infant's growth will be plotted on the WHO charts. The PIs will review growth charts at least twice monthly and then contact the PCPs by phone when appropriate after each individual is closely evaluated as described below. For any individual child that meets initial screening criteria for growth concerns, numerous factors will be considered in determining whether the child's growth is problematic and/or related to study interventions. Examples of such factors include genetic potential based upon parental size, the participant's linear growth, feeding mode (breast milk vs. formula), and interval illnesses. If either the primary care provider or the study investigators believe that it is possible that these growth patterns are a negative result from study participation, the child will be withdrawn from the study.

Expected Rates for Failing Screening Criteria for Adverse Events Potentially Related to Study Interventions

Using standard population distributions, it is expected that 5% of children will be below the 5th percentile on the weight-for-age growth chart. For downward crossing of two major centile lines, it is expected that this will occur with some frequency given the well-established phenomenon of regression to the mean, which suggests that those born at the higher percentiles have a higher probability of moving downward to the population mean. The results of our pilot study suggest that 15% of participants may experience this, and that will serve as the cut-off alarm value for this study. Combined, these two screening criteria allow for up to a 20% adverse event rate for those events potentially related to the study interventions.

b. *Describe what data are reviewed, including safety data, untoward events, and efficacy data.*

As described above, adverse events reports and infant growth will be monitored.

c. *Describe how the safety information will be collected (e.g., with case report forms, at study visits, by telephone calls with participants).*

We plan to collect adverse event data from the intervention and control groups as they occur using adverse event reports.

d. *Describe the frequency of data collection, including when safety data collection starts.*

Safety data collection will begin immediately after the study commences. We plan to collect adverse event data from the intervention and control groups as they occur, and will report them quarterly. As noted above, the PIs will review growth charts at least twice monthly.

e. *Describe who will review the data.*

Because our proposal includes vulnerable populations (i.e., African American infants and their mothers), we will include a Data Safety Monitoring Board (DSMB). The DSMB will be chaired by a pediatrician with expertise in patterns of normal and abnormal growth in infants (this individual will also serve as the Safety Officer for the study), and will also include a biostatistician as well as researchers with expertise in issues relating to African American families, infant and family behavioral interventions, and maternal health and infant development.

f. *Describe the frequency or periodicity of review of cumulative data.*

We will continuously monitor adverse event rates in all participants. In consultation with PI Stansfield, PI Lavner will have primary responsibility for reporting Adverse Events, Serious Adverse Events, and Unanticipated Problems to the AU IRB, the study's DSMB, and the NIH/NIDDK as required. PI Birch will have secondary responsibility for reporting.

Safety reports will be sent to the study statistician, the PIs, and the Safety Officer/Chair of the DSMB quarterly. At least once per year, the DSMB will convene to review study progress and issues related to the safety of the study interventions. Meetings may be more frequent than once per year if the need for such meetings becomes apparent to the Safety Officer, PIs, IRB, or NIH/NIDDK. The project coordinator will be responsible for assembling the data and producing these reports in conjunction with the study statistical team, as well as assuring that all parties obtain copies of these reports.

g. *Describe any conditions that trigger an immediate suspension of the research.*

Stopping Rules.

As outlined above, we will continuously monitor adverse event rates in all participants. The safety officer, together with the study investigators, will alert the IRB and the NIH if a greater than or equal to 20% adverse event rate potentially due to study interventions should occur in the treatment group or if significant differences between treatment groups occur.

As described in detail above, we will closely monitor rates of insufficient growth for individual infants and by study treatment group. In addition to individual level monitoring, the study statistician will analyze rates of insufficient growth by treatment group on a quarterly basis. If there are significantly more children with inadequate growth in the intervention group at any point, these data will be presented to the AU IRB, the study's DSMB, and NIH/NIDDK so that a decision can be jointly made as to whether the trial needs to be suspended.

We acknowledge that there are other situations that could occur that might warrant stopping the trial. We have a section on the safety report entitled 'Other situations that have occurred since the last safety report that warrant discussion' to allow for communication of concerns to the study PIs, statistician, and the Safety Officer.

9. Withdrawal of Subjects ☐ N/A

a. *If applicable, describe anticipated circumstances under which subjects will be withdrawn from the research without their consent.* ☐ N/A

Any participant with a physician-diagnosed serious adverse event related to the study interventions will be discontinued from the study. Should insufficient weight gain be detected and the cause of this weight gain is potentially related to the study interventions as determined by the PIs after consultation with the participant's primary care provider, the participant will be discontinued from the study. Other non-minor concurrent illnesses or major changes in the family social environment would also lead to discontinuation as determined by the investigators alone or in consultation with the PCP.

b. *If applicable, describe any procedures for orderly termination.* ☐ N/A

In the event that a subject is withdrawn from the research without their consent (whether due to unforeseen change in meeting inclusion criteria or an adverse event), all contact with subject will be ceased. Subjects will receive monetary compensation only for those appointments and study requirements met until the date of withdrawal. Any materials provided (bassinet, intervention materials) as part of the study procedures will remain

with the subject. Only if agreed to at the time of consent, data collected for that subject dyad until the date of withdrawal will remain part of the study data and included in researcher analysis where possible.	
c. *If applicable, describe procedures that will be followed when subjects withdraw from the research, including partial withdrawal from procedures with continued data collection.*<br><br>In the event when subjects withdraw from the research, all contact with subject will be ceased. Subjects will receive monetary compensation only for those appointments and study requirements met until the date of withdrawal. Any materials provided (bassinet, intervention materials) as part of the study procedures will remain with the subject. Only if agreed to at the time of consent, data collected for that subject dyad until the date of withdrawal will remain part of the study data and included in researcher analysis where possible.	☐ N/A

10. Risks to Subjects

a. *List the reasonably foreseeable risks.*<br><br>*Potential risks*. All participants will continue to receive their standard care from their medical providers. As described above, we will contact infants' primary care providers to notify them of the dyad's participation in the study. Our previous research found no increased risk of adverse events, or specifically failure to thrive, among those infants randomized to receive the multi-component prevention program. Given that the proposed study is focused on infant sleep and soothing, rather than focused on feeding, and is of significantly shorter duration than our previous multi-component trials, there is extremely minimal risk of insufficient weight gain and/or failure to thrive in the experimental group.	
b. *If applicable, describe any costs that subjects may be responsible for because of participation in the research.*	■ N/A
c. *If applicable, describe risks to others who are not subjects.*	■ N/A

11. Potential Benefits to Subjects

Describe the potential benefits that individual subjects may experience from taking part in the research.

Participants may experience improved health outcomes such as improved sleep and soothing, and healthier weight status during the first 16 weeks of life. All participants may benefit from sleep safety and those in the safety control arm may benefit from the home-based safety interventions. Childhood obesity may be prevented in some subjects in the study. There may also be positive effects for mothers' individual and relationship functioning.

12. Confidentiality

Describe the procedures for maintenance of confidentiality.

Subject confidentiality will be a priority for the research team, with prudent measures taken to ensure security of data and personal information. Upon enrollment, subjects will be assigned a study ID number which will serve as a code associated with all collected data. This code will be linked to the subject in only one document, the project directory, which will be password protected using an approved data management software system. Only pertinent study team members (PIs, coordinators) will have access to any and all data using a secure internet connection. Electronic and paper versions of de-identified data will be stored separately from any files that may contain subject names.

13. Consent Process

If you are obtaining consent of subjects describe the consenting process.

At the time of recruitment (AU newborn nursery), qualified mothers will be asked to read and sign a document indicating informed consent for herself and a separate parental permission document indicating consent for her infant to participate. The approved consent/permission documents will detail all study procedures and address incentives, risks, benefits, confidentiality and potential withdrawal. Mothers will have the opportunity to have any questions answered by the recruitment coordinator or PI prior to making the decision to enroll.

14. Compensation for Research-Related Injury

This section is not required when research involves no more than Minimal Risk to subjects. ☒ **N/A**

a. *Describe the available compensation in the event of research related injury.*

15. Resources Available ☐ N/A

a. *Describe the availability of medical or psychological resources that subjects might need as a result of an anticipated consequences of the human research.*

Although we do not anticipate any negative consequences of this research, all participants will receive a handout at the first visit with a list of community resources, including public health, mental health, food banks, shelters, division of labor, etc.

b. *Describe your process to ensure that all persons assisting with the research are adequately informed about the protocol, the research procedures, and their duties and functions.*

All study team members will be trained according to individual responsibilities with respect to the project and managed by the PIs and the Project Coordinator. Regular meetings of the core study team from each site (AU and UGA) will ensure efficient communication and management of personnel. All study team members will maintain appropriate training certifications.

Consistent with protocols used in the pilot trial and efficacious programs for rural African American families, the Community Research Associates (CRAs) who will serve as the Sleep-SAAF intervention home visit facilitators are African American community members from communities similar to the ones where the intervention will be implemented. However, CRAs will not reside in the same communities in which they are delivering the intervention. CRAs must have a minimum education level of high school graduation and are selected based on information from interviews and references that attests to their communication skills, engaging personalities, and ability to implement a structured program with fidelity. CRAs will receive 30 hours of training on the implementation of curriculum activities, guided practice in delivering and pacing curriculum segments, and facilitator self-care. They will work from a detailed manual that describes all facets of program delivery. Didactic material, role-playing exercises, and modeling will be used to teach the protocol for each session. Prior to implementation, CRAs must demonstrate mastery of the curriculum by passing a written test covering the session material. To ensure ongoing quality control and fidelity, CRAs will meet with the Project Coordinator and/or Intervention Coordinator weekly.

Protocol Title: Sleep-Safe: A Strong African American Families Study

Principal Investigator: Brian Stansfield, MD

1. Objectives

Describe the purpose, specific aims, and hypothesis:

Rapid weight gain in infancy is a risk factor for obesity and comorbidities later in the lifespan. This project is designed to test whether teaching mothers prompt, contingent, developmentally appropriate responsive parenting skills to soothe infants and promote adequate sleep can reduce rapid weight gain among African American infants living in the rural South. **Specific Aim 1: To assess the effects of responsive parenting, focused on infant sleep and soothing, on infant weight and reducing rapid weight gain from birth to 16 weeks among African American infants.** We hypothesize that infants in the sleep/soothe condition will have lower weight outcomes (i.e., BMI z scores, weight-for-length z scores, weight-for-age z scores) at age 16 weeks and gain weight more slowly compared to infants in a safety control condition. We consider whether weight-related factors (e.g., maternal pre-pregnancy BMI, gestational weight gain, intent to breast or formula feed, maternal smoking) moderate intervention effects. **Specific Aim 2: To assess effects of responsive parenting on parental and infant behaviors, and whether these mediate effects on infant growth.** We hypothesize that compared with control parents, parents in the sleep/soothe condition will show increases in responsive parenting, parenting self-efficacy, and the use of alternatives to feeding to soothe (white noise, pacifier, etc.), and decreases in feeding to soothe. Over time, infants in this condition will have longer sleep bouts and fewer feedings. These outcomes will be linked to healthier patterns of weight gain from birth to age 16 weeks, fully accounting for group differences. **Specific Aim 3: To examine moderation of intervention effects by individual and contextual factors.** We examine whether individual and contextual factors common among African American mothers moderate the effectiveness of the sleep/soothe intervention, informing development of future multi-component programs.

2. Background

Describe the background and rationale for the study:

This project is in response to the call for research to understand factors in infancy and early childhood that affect obesity development. Racial disparities in overweight among African American children compared to White children begin early in development and continue throughout the lifespan. During infancy, African American children have elevated rates of rapid weight gain and greater prevalence of high weight for length. Rapid weight gain is one of the most consistent risk factors associated with later overweight and obesity, showing associations with later overweight and obesity among toddlers, children, and young adults. Despite these well-established associations and the clear need for early intervention, there has been limited attention to developing interventions to prevent rapid weight gain among African American infants within underserved, low SES contexts. Accordingly, we propose to test whether teaching mothers prompt, contingent, developmentally appropriate responsive parenting skills to soothe infants and promote adequate sleep can reduce rapid weight gain among African American infants living in the rural South. Our examination of sleep and soothing evaluates a component of early obesity prevention programs that is promising, attractive to potential participants, and that may be particularly potent for African American infants in underserved, low SES contexts, making it the natural starting point for efforts to apply responsive parenting interventions in this high-risk context.

Findings from our two previous RCTs have provided evidence for efficacy of a multi- component responsive parenting (RP) program among middle income, predominantly White mother-infant dyads. Our team tested whether a multi-component RP program, delivered using home visits and including guidance on infant sleeping, soothing, active social play, and feeding, including the transition to table foods during the first year of life, could reduce rapid weight gain during infancy and overweight in early childhood. Relative to a home safety control, infants randomized to the RP program demonstrated significant differences in sleep behaviors at 8-, 16-, and 40-weeks, including longer nighttime sleep duration, had more consistent bedtime routines and earlier bedtimes, and were more likely to self-soothe to sleep without being fed. Relative to control, infants of parents receiving the RP program gained weight more slowly in the first months after birth, had lower weight-for-length percentiles at 1 year, and were less likely to be overweight at 1 year.

Shorter sleep duration is more prevalent among African Americans and is related to multiple adverse health outcomes including obesity. In infancy, sleep duration and feeding are tightly linked; sleep bouts tend to be interrupted by feeding; higher feeding frequency is associated with shorter sleep duration. The responsive parenting sleep/soothe intervention has the potential to alter parenting and infant behaviors (sleep, soothing, and feeding), directly affecting rapid weight gain and ultimately long-term weight outcomes, by promoting appropriate, prompt, and contingent soothing responses to infant crying. Focus groups with African American mothers suggest that the intervention will be well received, but the potential for moderation of effectiveness by individual (depressive symptoms) and contextual (poverty, relationship support) stressors common to African American mothers is currently unknown. Examining these factors as they relate to maternal response and rapid weight gain among infants will increase our understanding of how stress affects high-risk samples, potentially identifying additional intervention foci to be targeted in a subsequent multi- component program optimized for this high-risk population. Likewise, testing the mediational model that has guided this research to date and determining its applicability in this sociocultural context is an essential step in optimizing future interventions.

Our conceptual model is shown below. As shown in the Figure, we argue that a responsive parenting sleep/soothe intervention (Sleep SAAF) has the potential to alter parenting behaviors (responsive parenting, parenting self-efficacy, alternatives to feeding to soothe) and infant behaviors (sleep, soothing, and feeding). These in turn affect rapid weight gain and ultimately long-term weight outcomes. However, the effectiveness of this intervention may be affected by individual and contextual stressors common to African American mothers, including depressive symptoms, socioeconomic and race-based stressors, and a challenging interpersonal context.

Figure 1. Conceptual Model: Early Factors Affecting Rapid Weight Gain among African American Infants

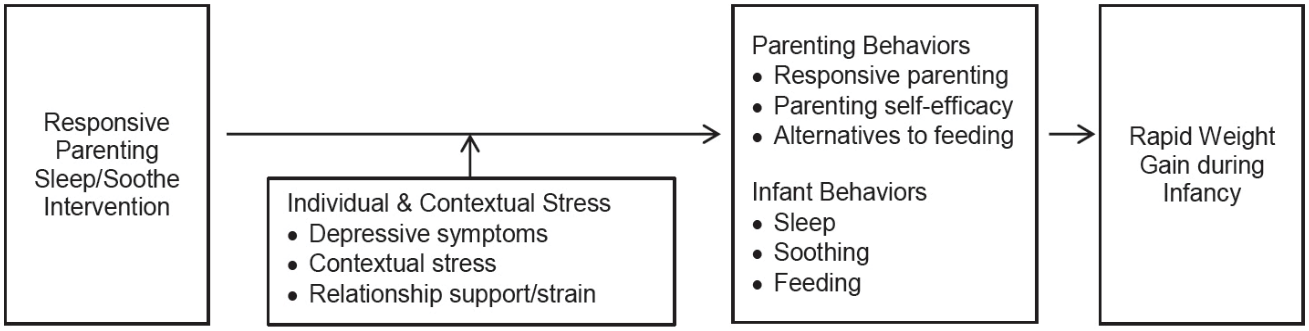

3. Inclusion and Exclusion Criteria

List the inclusion/exclusion criteria:

A total of 375 African American mother-infant dyads will be recruited from AU's newborn nursery. Co-participation of the mother-infant dyad is a study requirement. Paternal involvement is voluntary, but not necessary. If fathers are interested in participating, they will sign separate consents.

INCLUSION CRITERIA

Eligible mother-infant dyads for this trial will meet the following criteria:
1) full-term infants (≥ 37 0/7 weeks gestational age), apparently healthy and without significant morbidity
2) late pre-term infants (between 34 0/7 and 36 6/7 weeks gestational age), apparently healthy and without significant morbidity
3) singleton infant
4) nursery/NICU/maternity stay of 7 days or less
5) Mother at least 18 years of age
6) Mother self-identifies as African American
7) Mother is primiparous
8) Parental permission may be obtained from one parent to allow mothers who are 16 or 17 years of age to participate. Minor assent will also be obtained from 16 or 17 year old mothers at the time of enrollment. Mothers who are enrolled as minors but who become adult aged (18 years) while enrolled will be re-consented as adults before continuing in the study.

C. EXCLUSION CRITERIA

(not explicitly obvious from the inclusion criteria)
1) non-English speaking
2) infant birth weight <2500 grams (for full-term infants)
3) infant birth weight <2100 grams (for late pre-term infants)
4)presence of a congenital anomaly or neonatal physical or metabolic condition that significantly affects a newborn's feeding (e.g. cleft lip, cleft palate, metabolic disease)
5) any major maternal morbidities, pre-existing condition that would affect postpartum care or her ability to care for her newborn (e.g., narcotic drug use: heroin, cocaine, meth, pain pills, etc; on chemotherapy; uncontrolled MS; uncontrolled depression causing social service contact).
6) plan for newborn to be adopted
7) plan to move from area within four months of delivery
8) residence further than 75 miles from Augusta, GA
9) current or prior residence with another individual who participated or is currently participating in this study

4. Number of Subjects/Records/Samples Collected

Indicate the total number of subjects to be accrued/records reviewed/samples collected across all sites:

Up to 375 mother-infant dyads and up to 375 fathers will participate in the study. All mothers will complete a series of questionnaires at enrollment and at 4 follow-up time points (study procedures described below). AU medical records will be used to gather selected demographic information on mothers (race, maternal age, gestational age, weight at delivery, address) and infants (date of birth, weight and length at delivery). Fathers will be invited to participate by the recruitment coordinator if they are present at the hospital, or by the study team if they are present at the first home visit (1 week postpartum). Fathers will a sign separate consent form.

The study will be conducted over 4 years. The table below details the number of target participants per year:

Year	Mother-Infant Dyads	Fathers (Max #)	Research Interactions
Year 1	50	50	200
Year 2	125	125	500
Year 3	125	125	500
Year 4	75	75	300
**Total**	**375**	**375**	**1500**

5. Recruitment Methods

Describe when, where, and how potential subjects will be recruited:

Recruitment of 375 African American mothers and their infants will take place at Augusta University Medical Center in the Children's Hospital of Georgia's mother/baby nursery, when potential subjects are newly postpartum. A dedicated recruitment coordinator will be hired (AU staff) and trained to identify (via active medical chart data), screen, and enroll qualified subjects.

With approved Waivers of Consent and HIPAA Authorization, the recruitment coordinator will have access to electronic medical records systems and will complete the Sleep-Safe Screening and Eligibility Form Part 1 in order to pre-screen mothers and infants. The recruitment coordinator will approach those who match basic inclusion/exclusion criteria as detailed in Section 3 of this document. The recruitment coordinator will deliver an approved verbal script to pre-screened mothers to determine interest in participation. Interested mothers will respond to remaining screening questions (Screening and Eligibility Part 2) in order to confirm eligibility. Qualified and interested participants will then complete informed consent and move through the enrollment process with the recruitment coordinator.

6. Procedures Involved

a. *Describe the procedures involved to include those procedures that are standard evaluation and/or care and those that are solely for research purposes:*

All procedures are solely for research purposes. No contact and reduced contact interactions may be implemented in response to COVID-19. All interactions will include use of Personal Protective Equipment (PPE) and sanitization guidelines for both research associates and participants. A health screening will be administered prior to each visit and visits will be rescheduled if COVID-19 symptoms are endorsed.

Enrollment at AU Hospital Newborn Nursery (after delivery, Recruitment Coordinator):

1. Obtain maternal informed consent for mother-baby dyad
2. Obtain paternal informed consent for his own participation
3. Obtain selected demographic information (race, maternal age, gestational age, weight at delivery and (self-reported) pre-pregnancy weight) from the AU medical record system
4. Complete enrollment data collection forms
   a. Chart abstraction form (coordinator completed)
   b. Contact form
5. Measure weight (kg) and height (cm) of mothers and fathers (if father not present, will attempt to measure at subsequent visit)
6. Measure infant anthropometry: length (cm), weight (g), head circumference (cm), and mid-thigh circumference (cm).
7. Schedule the first research visit at the home for 1 week postpartum
8. Provide participants with overview of study safety guidelines

Home Visit 1: (Home, 7-10 days postpartum, Community Research Associate (CRA))

1. Randomize and provide appropriate introductory materials (intervention or control) to mother-infant dyads
2. Complete questionnaire measures
3. Measure infant weight and length
4. Measure maternal weight
5. Measure paternal weight

Home Visit 2: (Home, 3 weeks, CRA)

1. Deliver intervention or control group program
2. Complete questionnaire measures
3. Measure infant weight and length
4. Measure maternal weight
5. Measure paternal weight

Home Visit 3: (Home, 8 weeks, CRA)

1. Deliver intervention or control group program
2. Provide mothers with activity monitors/diaries (to be picked up by study staff after 7 days' use)
3. Complete questionnaire measures
4. Measure infant weight and length
5. Measure maternal weight
6. Measure paternal weight

Telephone call: (12 weeks, CRA)

1. Scheduled phone call with mothers to check in and remind of 16 week assessment

Home Visit 4: (Home, 16 weeks, CRA)

1. Complete questionnaire measures
2. Measure infant weight, length, head circumference, and mid-thigh circumference
3. Measure maternal weight
4. Measure paternal weight
5. Complete questionnaire to determine interest in participation in one year follow-up visit and to update contact information

No-contact Home Visit 4: (Home, 16 weeks, CRA)

A no-contact visit will be offered to the 24 mother-infant dyads active in the study but unable to complete study procedures due to COVID-19 restrictions. After a precautionary health screening and providing instructions to participant, a research associate will place sanitized materials (iPad, infant scale, adult scale) outside the door to the home. The participant will then retrieve these materials and complete study procedures inside the home. The research associate will be available by phone to assist if needed, and will retrieve materials once replaced outside by participant. There will be no face-to-face contact between the research associate and the participant.

1. Complete questionnaire measures
2. Measure infant weight
3. Measure maternal weight
4. Complete questionnaire to determine interest in participation in one year follow-up visit and to update contact information

Researcher will request permission at the time of consent to photograph mother and/or infant during a research visit. These photos would be used in conjunction with presentations or publications related to this study.

**** Table 1 provides additional details on scheduled assessments at each time point. We provide additional information on the intervention and control group materials in the following section on study design.* Participating fathers will also complete measures listed in parent outcomes, stress-support moderators, and demographics. *Refer to Appendix 4 – Measures****

Table 1. Evaluation Schedule of Assessments and Measures by Time Point				
	1wk (R)	3wk (I)	8wk (I/R)	16wk (R)
Anthropometry				
Anthropometry - Baby	X	X	X	X
Anthropometry - Mother		X	X	X
Anthropometry - Father		X	X	X
Sleep Outcomes				
Parent report of infant sleep - Brief Infant Sleep Questionnaire (BISQ)		X	X	X
Parent report of own sleep – Insomnia Symptom Questionnaire (ISQ)	X		X	X
Maternal Actigraphy			X	
Infant Actigraphy			X	
Infant Soothe and Feeding				
Infant soothing – Babies Need Soothing			X	X
Infant temperament - Infant Behavior Questionnaire				X
Feeding frequency - Babies Need Feeding		X	X	X
Baby's eating behavior – Baby Eating Behavior Questionnaire (BEBQ)				X
Parent Outcomes				
Parenting self-efficacy - Karitane Parenting Confidence Scale			X	X
Maternal feeding practices and beliefs – Infant Feeding Style Questionnaire (IFSQ)			X	X
Maternal depression – CES-D	X		X	X
Self-reported physical health				X

Child Safety Outcomes				
Safe sleep practices – Fowler	X			X
Child safety practices – Framingham Safety Survey				X
Stress-Support Moderators				
Maternal Personality – Mini IPIP	X			
Family Background – Risky Families Questionnaire	X			
Financial strain/employment – Assorted questions	X			
Discrimination – Schedule of Racist Events	X			
Romantic relationship characteristics – Assorted questions	X		X	X
Co-parent relationship characteristics – Coparenting relationship scale				X
Social support – Social Provisions Scale	X			
Family routines - Confusion, Hubbub and Order Scale (CHAOS)			X	
Demographics and Health History				
Family demographics, maternal/infant health	X			
Intervention-Related Variables				
Co-parent involvement in intervention		X	X	
Implementation quality		X	X	

Infant Growth

Infant weight and length. At each home visit, infant weight and recumbent length/height will be measured by research staff trained in obtaining anthropometrics, and will be used to calculate weight-for-length and BMI percentile and BMI Z-score based on percentiles for age and sex established by the WHO (for outcomes <2 years) and CDC (for outcomes 2 years and up). Weights will be obtained using Medela Baby Weigh II infant scales. Recumbent lengths will be obtained in triplicate with Seca Model 416 recumbent length board. Head circumference will be measured using Seca Model 212 band and mid-thigh circumference will be measured using a flexible anthropometric measuring tape.

Sleep Outcomes

Parent report of infant sleep. The validated Brief Infant Sleep Questionnaire (BISQ) will be used to assess sleep. This survey assesses infant sleep location, before-bedtime activities, and sleep patterns. Sleep duration is divided into nighttime (7 pm–7 am) and daytime (7 am–7 pm) and is reported in hours and minutes. Selected questions capturing sleep duration (day and night), location, and night feedings will also be included.

Parent report of own sleep. Parents will respond to the Insomnia Symptom Questionnaire (ISQ), a validated measure used to estimate sleep duration and quality.

Maternal Actigraphy. We will also use actigraphy data from mothers to provide an objective assessment of maternal sleep that complements maternal self-report of infant sleep. Mothers will wear an accelerometer (Philips Respironics Actiwatch 2) on the wrist of the non-dominant hand for a 7-day period following the 8-week assessment and complete a diary to record sleep and device use..; monitors will be retrieved by CRA.

Infant Actigraphy. Actigraphy data from infants will provide an objective assessment of infant sleep that complements maternal self-report of infant sleep. Infants will wear an accelerometer (Ambulatory Monitoring, Inc, MicroMini Motionlogger) on the ankle of for a 7-day period following the 8-week assessment, mothers will complete a diary to record sleep and device use; monitors will be retrieved by CRA.

Infant Soothe and Feeding Outcomes

Soothing practices. The Babies Need Soothing scale from the Baby's Basic Needs Questionnaire measures the extent to which parents use feeding for reasons other than in response to hunger, such as to soothe, calm, or control behavior. This measure assesses: (1) reasons for crying, (2) frequency and effectiveness of soothing techniques, including feeding, (3) foods used to soothe, and (4) contexts where food to soothe is used.

Infant temperament. Infant temperament will be assessed using the validated Infant Behavior Questionnaire. Infant difficultness, characterized by fussiness and difficulty soothing, has been related to rapid weight gain, body composition, and increased obesity risk.

Feeding frequency. The Babies Need Feeding scale from the Baby's Basic Needs Questionnaire was used in our previous studies to examine current feeding mode (breast milk and/or formula), percent of daily feedings that are breast milk or formula, as well as the use of bottle feeding of formula or human milk.

Baby's eating behavior. The Baby Eating Behavior Questionnaire (BEBQ) will be used to measure maternal perception of infant hunger and satiety.

Parenting Outcomes

Parenting self-efficacy. The validated 15-item Karitane Parenting Confidence Scale measures perceived parental self-efficacy in the parents of infants aged 0-12 months. This measure will allow us to test whether parents' feelings of self-efficacy mediate intervention effects and whether parenting self-efficacy relates to the behavioral variables of interest.

Maternal feeding beliefs and practices. The Infant Feeding Styles Questionnaire is a validated self-report instrument that assesses maternal feeding beliefs and behaviors. It was developed specifically among low-income African American mothers.

Maternal depression. We will assess maternal depressive symptoms with the widely used Center for Epidemiological Studies Depressive Scale.

Self-reported physical health. Participants will rate their physical health with selected items from the SF36 from the Medical Outcomes Study.

Child Safety Outcomes

Safe sleep practices. We will assess safe sleep practices using a 6-item measure evaluating the frequency of sleep practices such as how often the baby sleeps with toys, with an adult, and on her back.

Child safety practices. The Framingham Safety Survey for the first year of life will be administered to assess high-risk behaviors or conditions reported by parents that occur in the home. It will be used to evaluate the impact of the Child Safety Control. This survey was adopted by the AAP and is a screening tool for injury prevention.

Stress-Support Moderators

Maternal personality. Personality will be assessed using the mini-IPIP (Donnellan, Oswald, Baird, & Lucas, 2006), a validated measure of the Big Five personality traits.

Family background. Parents' experiences during childhood will be assessed with the widely-used Risky Families Questionnaire (Taylor, Lerner, Sage, Lehman, & Seeman, 2004).

Financial strain/unemployment. Socioeconomic stressors will include a range of socioeconomic measures assessing individual and community level factors. Individual characteristics will include: (1) Socioeconomic Status, which combines education and income; and (2) Financial/Employment Situation, which will include multiple widely used scales that focus on financial adjustments, financial hardship, unmet needs, and employment. Community characteristics will include (1) Community Disadvantage, which will include the sum of 5 census variables used in previous research: % below poverty line, % single-parent families, % public assistance, % < high school education, and median family income; and (2) Social Isolation, which will include the combination of 3 census variables: % below poverty line, % living in the same house over years, and % housing occupied by owners.

Discrimination/race-based stressors. We will include multiple measures of race-based stressors: (1) Discrimination, using the18-item long form of the widely used Schedule of Racist Events, which assesses both personal and vicariously-experienced discrimination, (2) Racial Segregation, using the % of racial groups in census tracts based on Massey and Denton's (1988) formula.

Romantic relationship characteristics. All mothers will report on their current romantic relationship status, including (a) married and cohabiting, (b) married but not cohabiting, (c) cohabiting, (d) romantically involved on a steady basis but not living together, (e) involved in an on-again and off-again relationship, and (f) single. Categories (d) and (e) were used in the Fragile Families and Child Wellbeing study to capture greater variability in the relationships of low-income families. Mothers who indicate that they are involved in a current romantic relationship will be asked whether this relationship is with the child's father or with another romantic partner.

Mothers who are involved in a romantic relationship will be asked to rate the following: (1) Relationship Satisfaction, using the 4-item Couples Satisfaction Index, (2) Conflict with Romantic Partner, using the 5- item Hostility Scale, (3) Warm, Caring, and Affectionate Behaviors, (4) Dedication Commitment, a 4-item scale assessing the desire to persist in the relationship despite obstacles, (5) Dissolution Risk, two items assessing thoughts of ending the relationship, and (6) Relationship Length and History, including how many times the current relationship has ended/resumed.

Coparent relationship characteristics. All mothers will also report on who is involved in coparenting (e.g., child's father, child's grandmother). Our feeding surveys will assess the proportion of feedings given by the mother as well as other caregivers. We will also assess aspects of the coparenting relationship (e.g., "My partner and I have different ideas regarding our child's eating, sleeping, and other routines") using selected questions from the Coparenting Relationship Scale.

Social support. Mothers will also be asked to rate the social support they receive from people other than their partner and/or coparent using the Social Provisions Scale.

Family routines. We will assess the extent to which parents report that there is order and routine versus chaos and confusion in their home using selected questions from the Confusion, Hubbub, and Order Scale.

COVID-19 Impact Survey. This measure will be added to the study procedures for the 16 week (final) visit for 24 participants active during the hold on face-to-face research and will assess COVID-related psychosocial stressors and impacts. Following the resumption of new recruiting in summer 2020, this measure will be administered at the 1 week visit for all new participants.

Demographics and Health

Family demographics and maternal/infant health. Parent and demographic covariates will include: Pre- pregnancy BMI, smoking during pregnancy, gestational weight gain, maternal type 2 or gestational diabetes mellitus, maternal and paternal BMI assessed at the first research visit, income, education, and employment (work status, hours worked). Infant covariates will include: Infant weight at age 1 week (the first assessment) adjusted for gestational age, child sex, and feeding mode (predominantly breastfed if 80% or more of milk feedings were breastmilk, predominantly formula-fed if 80% or more of milk feedings with formula milk, or mixed-feedings).

Parent weight and height. Mothers' pre-pregnancy weight and weight prior to delivery will be obtained from chart abstraction, if available. Should these data be missing from charts, it will be collected via survey. Mother's height will be measured using stadiometer at AU and weight will be measured using a calibrated scale at AU. At all subsequent visits, mothers and fathers will again be weighed using calibrated scales.

Intervention-Related Variables

Coparent involvement in intervention. While paternal and/or coparent involvement in the study is not required for participation, fathers and/or coparents will be strongly encouraged to attend study visits so they too can receive the study intervention. We will collect data on their attendance and degree of participation in the intervention visits.

Implementation quality. Our previous evaluations gave us the opportunity to evaluate participants' responses to training and evaluation materials and this has led to revised and simplified training and assessment tools, suitable for those reading at a 6th grade level. As in our previous studies, we also include measures immediately after study visits to systematically assess the quality of intervention staff's implementation of the intervention. These measures will be completed by the Community Research Associates (CRAs) delivering the intervention (see below). These data will provide information on overall implementation quality in our sample and will also allow implementation quality to be investigated as moderator of intervention effects. We will use parallel measures with the child safety control group to assess implementation quality.

b. *Describe and explain the study design:*

The study design is a randomized controlled trial. This trial will deliver a responsive parenting intervention (Sleep SAAF) that builds upon our previously tested Soothe/Sleep curriculum to provide information on safe sleep practices, how to soothe, how to distinguish hunger from

other distress, how to promote self-soothing, and bedtime routines. The responsive parenting intervention will be compared to a safety control group. To promote uptake of program content, the programs will be delivered across 2 sessions at **3-weeks and 8-weeks postpartum**, allowing for increased dosage of this critical component. Assessments will be conducted at 1-week postpartum, 3 weeks postpartum, 8 weeks postpartum, and 16-weeks postpartum (final growth outcomes).

The intervention group will be compared to a child safety control group receiving an equal number of visits, which will be focused on child and sleep safety and should not affect infant weight gain. Both interventions will be delivered in the home setting by African American community research associates (CRAs) who are employed and trained by UGA's Center for Family Research (CFR). CRAs will also conduct data collection visits at participants' homes; when possible, the research visits (1 and 16 weeks) and the intervention visits (3 and 8 weeks) will be conducted by different CRAs. Self-report data will be gathered using a secure, web-based survey interface, Qualtrics. When preferred, audio computer-assisted self-interviewing (ACASI) software will be used to support data collection. ACASI elicits less social desirability bias and more accurate reports on sensitive issues than face-to-face interviews or written surveys. Video and audio enhancements guide respondents through the survey; literacy is not an issue. CRAs will receive extensive training on the implementation of curriculum activities. They will meet regularly with the Project Coordinator and Intervention Coordinator and participate in yearly refresher trainings. Our experienced data collection staff have successfully implemented these protocols in multiple projects.

All participants will receive information on proper infant sleep hygiene and creating a safe sleep environment, with a focus on "back to sleep," supine placement of the infant for sleep. We will use materials from NICHD's "Babies Sleep Safest on Their Backs: A Resource Kit to Reduce the Risk for SIDS in African American Communities", which was developed based on guidelines from the American Academy of Pediatrics. Guidelines include always placing baby on her back, placing on a firm sleep surface, not smoking around baby, and keeping baby's sleep area close to, but separate from, where mother and others sleep. For all participants, the sleep surface will be inspected and parents will be provided a bassinet if they do not already have one.

In addition to this information on safe sleep practices, participants in the intervention condition will receive guidance on responsive parenting, and the control group will receive additional content on child safety. Additional details are provided below.

Responsive Parenting Group. The Sleep SAAF responsive parenting intervention includes setting appropriate expectations regarding normal infant development and need for care during the first months of life, including infant sleep-waking, active alert behavior, crying, and feeding. Guidance on caregiving focuses on (1) how much sleep infants need and how to help them get it, (2) avoiding the use of feeding as the default response to infant crying, (3) how to discriminate hunger from other reasons for infant crying (too warm or too cold, too tired, diaper change needed), and (4) how to use alternative soothing strategies [including use of white noise, movement, side/stomach positioning while being held, non-nutritive sucking (pacifier), swaddling] to discriminate among causes of crying and to soothe a crying infant, as well as how to cope with crying. We will provide information about normal sleep patterns in infants. Guidance on how to help infants get the sleep they need will include establishing a bedtime routine that includes putting the infant to bed early, putting baby to bed drowsy but awake, dealing with night waking to promote self-soothing, and using "5Ss" to calm the baby. We will also discuss avoiding feeding the infant to sleep or putting the infant to bed with a bottle.

The Sleep/Soothe curriculum also includes some basic information on feeding, including normative information on how much and how often young infants typically eat, and on how to discriminate infant

hunger from other distress and to recognize when their baby is full. Because many caregivers assume that a fussy infant is hungry, feeding is often their first response to fussing and crying. During the intervention visits, intervention staff will work with parents of breastfeeding and formula feeding infants, as well as those feeding pumped breast milk, to recognize hunger cues (rooting, mouthing, bringing hand to mouth) and fullness cues (letting go of nipple, falling asleep, turning head away, interest in other things). Excerpts will be shown to parents to illustrate infant behaviors indicative of hunger and fullness. Expectations for typical feeding frequency during the day and night for breastfed and formula fed infants will be discussed. Parents will be given education on bottle sizes, milk/formula volumes, use of slow flow bottle nipples for infant under 4 months to prevent overfeeding or choking, and how to use fullness cues, rather than the amount of milk in the bottle, to determine when to terminate a feeding. Instructions will also advise parents to delay introduction of other beverages until age 6 months and to avoid addition of infant cereal to a bottle unless explicitly instructed to by a physician.

Having emphasized hunger and satiety cues, intervention staff will then provide training to parents in how to use alternative soothing strategies for non-hunger-related infant crying, including how to swaddle an infant, use of a pacifier, white noise, and use of movement and positioning. Intervention staff will discuss several methods, which can be used separately or in combination. Parents will be provided with video clips. During home visits, parents will be coached in the use of these strategies with their infant.

Child Safety Control Group. For the child safety control group, in addition to guidance on safe sleep (described above), several aspects of newborn safety will be discussed, guided by information from The Injury Prevention Program (TIPP) from the American Academy of Pediatrics as well as the Academy's guide for health supervision, *Bright Futures*. Following TIPP guidelines for ages 0-4, information will include prevention of car injuries, falls, burns, choking, and suffocation. To prevent car injuries, child safety seat installation will be reviewed. To avoid falls, parents will be reminded to never leave a baby alone on a changing table, bed, sofa, or chair. To prevent burns, parents will be encouraged to never carry a baby and hold a hot liquid or food at the same time; home smoke detectors and water heater will be evaluated. The information covered by the safety intervention is typically presented as part of standard pediatric office care, but will be delivered in a more hands-on fashion at the home visits for the child safety control group.

Intervention materials include materials from the American Academy of Pediatrics, Centers for Disease Control and Prevention, and other sources as well as videos and duplication. Other expenses include bassinets to be distributed to all study participants. Mothers receiving the intervention will receive group appropriate tools such as swaddle blankets, white noise machine, pacifiers, etc. Mothers in the safety control group will receive group appropriate materials, e.g., first aid kits, diapers.

Participating mothers will be provided with monetary incentives per visit as follows: 1 week: $50, 3 week $50, 8 week: $75, plus $25 at pickup of activity monitor, 16 week: $100. Total monetary incentive offered for each participant is $300. Each visit is expected to require 90-120 minutes and will include completion of project paperwork, documentation of informed consent, and the ACASI interview or the prescribed intervention activities. This incentive structure is in accordance with other IRB-approved participation incentives offered by CFR and is informed by feedback from community members.

c. *Describe the procedures performed to lessen the probability or magnitude of risks:*

As the current study is not blinded, the investigators will monitor for an increase in adverse events in each of the experimental conditions. Drs. Stansfield and Lavner will assume all responsibility for addressing adverse events. Because the study will include children, all IRB requirements for the protection of children will be fulfilled.

All participants will receive a handout at the first visit with a list of community resources.

All information obtained by project staff about the project participants including survey data and disclosures or observations during the assessment visits will be held strictly confidential.

Individual participant growth will be closely monitored by the investigators in order to identify growth failure. During each study visit, home visit staff will assess weight. There will be two initial screening criteria for growth concerns: (1) weight-for-age below the 5th percentile using growth charts from the WHO, and (2) downward crossing of a major percentile lines between any two study visits on the WHO weight-for-age growth chart statistically evaluated as a -0.67 Z-score change in order to provide a consistent measure across subjects. Each child's growth will be plotted on the CDC charts within one week following study visits to allow for identification of potentially concerning growth patterns. Dr. Stansfield will be immediately alerted to any concerning patterns (i.e., weight-for-age below the 5th percentile or downward crossing of two major percentile lines). After each child is closely evaluated, Dr. Stansfield will then contact the mothers by phone when necessary (i.e., when there is a potential adverse event related to infant growth), to discuss their infants' growth and whether a visit with the infant's primary care provider is necessary. If Dr. Stansfield is unable to reach the infant's mother and/or if the mother requests it, Dr. Stansfield will contact the infant's primary care provider directly. Dr. Stansfield will document these calls as they occur as a research note. Dr. Stansfield will review growth charts of all active study participants at least twice monthly.

For any individual child that meets initial screening criteria for growth concerns, numerous factors will be considered in determining whether the child's growth is problematic and/or related to study interventions. Examples of such factors include genetic potential based upon parental size, the participant's linear growth, feeding mode (breast milk vs. formula), and interval illnesses. If either the primary care provider or the study investigators believe that it is possible that these growth patterns are a negative result from study participation, the child will be withdrawn from the study.

Additional details regarding growth monitoring are included in Section 8 (Provisions to Monitor the Data to Ensure the Safety of Subjects) below.

d. *Describe the duration of an individual subject's participation in the study and the time involved:*

Mother-infant dyads are enrolled for a period of 16 weeks. Interactions include initial enrollment and 4 subsequent research and/or intervention visits to the family's home. Enrollment will take place within 24-72 hours of delivery (while the mother is admitted to the newborn nursery) and participation will continue until the infant is 16 weeks (not more than 30 weeks) of age. Initial enrollment procedures will take 60-90 minutes; each following study visit will take 90-120 minutes.

7. Data and Specimen Management

a. *Describe the data analysis plan, including any statistical procedures:*<br><br>Data analysis will be led by Dr. Lavner, who has expertise in the analysis of longitudinal data. Tianyi Yu, Ph.D., Assistant Research Scientist and Statistician at CFR, will provide statistical support for the conduct of analyses. All primary statistical analyses will invoke the intent-to-treat paradigm, analyzing data based on randomized assignment. Missing data will be handled using full-information maximum likelihood methods, which use all available information to estimate parameters, making this approach more efficient and less biased than other methods when data are missing at random. All models considered allow for the inclusion of relevant covariates. Covariates to be considered in refining the analyses, in addition to those described in detail in the proposed analyses below, include demographic factors (e.g., maternal employment and hours worked, gestational diabetes), weight-related factors (e.g., maternal pre- pregnancy weight/BMI, gestational weight gain, maternal smoking), and intervention-related factors (e.g., implementation quality, coparent involvement in intervention). We will also examine (1) infant sex as a covariate, given different growth charts for boys and girls and (2) feeding mode as a covariate, given differences in weight gain between breastfed and formula-fed infants.<br><br>***Specific Aim 1: The Effects of Sleep SAAF on Infant Weight and Rapid Weight Gain***. In Specific Aim 1 the primary outcomes are infant weight and changes in weight. BMI is generally assumed to be the standard growth measure for assessing obesity-risk in children age 2 years and older, but there is not one universally accepted measure for children younger than age 2 years. Recent data suggests that infant BMI at age 2 months is more strongly associated with obesity at age 2 years than is weight-for-length. Accordingly, BMI z scores will be our primary outcome, but we will also consider models that examine a range of other weight outcomes, including weight for length z-scores, percentiles, and weight for age z-scores; we anticipate that change across different indices of weight gain will be highly correlated and lead to similar conclusions in tests of program impact as well as tests of potential moderators and the hypothesized mediational model.<br><br>First, we will examine differences in BMI and other weight outcomes at 16 weeks between the Sleep SAAF group and the control group using a linear mixed-effects model. The linear mixed-effects model will include intervention group and infant weight at age 1 week adjusted for gestational age. These analyses will test whether the Sleep SAAF group and the control group differ in their weight outcomes at age 16 weeks, which has previously been linked to child obesity risk at age 2 years.<br><br>Next, we will consider growth models that examine rate of change in weight from birth to 16 weeks. We will conduct growth curve analyses using hierarchical linear modeling to test whether the Sleep SAAF and control groups differ in their rate of change in weight over time. The model will include intervention group, time of growth measure, and infant weight adjusted for gestational age. These analyses will evaluate whether the Sleep SAAF group shows less of an increase in weight over time compared to the safety control group.<br><br>As an additional test of rapid weight gain, weight gain z-scores will be calculated for each measurement subsequent to the first intervention session at age 3 weeks (8 weeks and 16 weeks). Rapid weight gain scores will be calculated as an increase in weight-for-	☐<br>N/A

age z-score > +0.67, as described by Ong and Loos. This increase corresponds to crossing adjacent major centile lines on the standard CDC growth charts. The analysis for these rapid weight gain scores will be a repeated measures analysis, assessing the intervention effects on rapid weight gain scores from age 3 weeks up to age 16 weeks. The analysis will use generalized estimating equations (GEE) with a logit link, an extension of logistic regression which allows for repeated measurements on binary data, and will include infant weight adjusted for gestational age as a covariate. Because it is unlikely that within an 16-week period we will have many infants who meet the criteria for rapid weight gain based on the upward percentile crossing approach, we will also calculate conditional weight gain scores (CWG) following the method of Griffiths and colleagues, as described in our recent work.

Specific Aim 2: The Effects of Sleep SAAF on Parental and Infant Behaviors that Mediate Effects on Infant Growth. In Specific Aim 2 the primary outcomes are parental behaviors (parental self-efficacy, sleep/soothing practices, feeding practices, family chaos) and infant behaviors (sleep, soothing, and feeding) targeted by the intervention that may mediate effects on infant growth. We examine these effects in several steps.

First, we will examine differences between the Sleep SAAF and control groups in these outcomes at two time points post-intervention (8- and 16- weeks postpartum) using the linear mixed-effects model described above. The linear mixed-effects model will include intervention group and infant weight adjusted for gestational age. These analyses will test whether the Sleep SAAF group and the control group differ in these behaviors post-intervention and will be used to identify variables for the formal test of mediation.

Second, we will examine whether parent and infant behaviors mediate infant weight outcomes. We will test these hypotheses using structural equation modeling (SEM). The first step in demonstrating mediation is to establish the effects on mediating and distal outcomes, as we described above. We then specify mediators as indirect effects in a path model. Intervention condition will be dummy coded and specified as a predictor of infant and parental behaviors, which in turn predict infant weight and weight gain. The significance of the mediating process will be tested using the Sobel or bootstrapping methods.

Specific Aim 3: Moderation of Intervention Effects by Individual and Contextual Factors. In Specific Aim 3 we consider whether individual (depressive symptoms) and contextual (socioeconomic stressors, race-based stressors, romantic relationship characteristics, coparent relationship characteristics, social support) factors at 1 week postpartum moderate program effects. These factors will be included in the analyses described above to determine whether they moderate intervention effects on weight outcomes (Specific Aim 1) and parental and infant behaviors (Specific Aim 2).

As an exploratory analysis, we will also evaluate whether the individual and relationship variables differ between the Sleep SAAF group and the control group at 16 weeks postpartum. Doing so will allow us to examine whether improving infant sleep and soothing has secondary benefits for mothers' individual and relationship functioning.

b. *When applicable, provide a power analysis:* ☐ N/A

Power

The primary outcomes for this study will be BMI z scores at 16 weeks (Specific Aim 1) and parent and infant behaviors at 8 weeks and 16 weeks (Specific Aim 2). We used G Power 3.1.9.2 to estimate the smallest intervention effect size we can detect with 80% power, a 5% two-tailed Type 1 error rate, and two groups of 175 each (conservatively assuming 10% attrition over the course of the study). We can achieve 80% power with an effect size *d* of 0.34. Our recent research showed an effect size of approximately 0.4 for infant nighttime sleep duration at 8 weeks and for infant conditional weight gain at 6 months, suggesting that we will have sufficient power in our sample to detect intervention effects.	
c. *Describe how data and specimens will be handled:*	☐ N/A
i. *What information will be included in that data or associated with the specimens?*<br><br>Subject data, including measurements, questionnaire data and intervention evaluation data will be maintained for analysis of study outcomes. The staff at AU and UGA will coordinate the creation of all paper and electronic data collection and management forms that will be distributed to research subjects and subsequently returned to researchers. Aside from consent/permission documents and contact information, all study data will be de-identified to ensure confidentiality. Upon enrollment, subjects will be assigned a study ID number which will serve as a code associated with all collected data.	
ii. *Where and how data and/or specimens will be stored?*<br><br>Electronic participant data will be stored on password protected computers, with all files also protected by password. Original and all copies of paper or hard copy data files will be stored on site (AU or UGA) in locked file cabinets. De-identified data will be stored separately from any files that may contain subject names. The project directory will be the only document containing a key code linking subjects to study ID numbers; this directory will be password protected. Only pertinent study team members (PIs, coordinators) will have access.	
iii. *How long will the data and/or specimens be stored?*<br><br>At the time the study is completed and closed, all identifiable data (project directory) will be destroyed. However, subjects who wish to take part in long-term follow-up will agree in writing (consent form) to allow researcher to maintain identifiable data until follow-up is complete, or until they decide to discontinue participation. De-identified data will to be stored and analyzed indefinitely.	
iv. *Who will have access to the data or specimens?*<br><br>Only pertinent study team members (PIs, recruitment and project coordinators) will have access to the project directory. Other study team members (per IRB record), including graduate students, CFR team and statistical analysis team, will have access to raw data to be entered and analyzed per the study outcomes.	
v. *Who is responsible for receipt or transmission of the data and/or specimens?*	

Only pertinent and appropriate (as specified in section iv.) study team members will receive or transmit collected data for storage and analysis. Data will be entered via secure and password protected Qualtrics software on laptop computers or tablets, and will subsequently be transmitted to secure AU or UGA facilities as soon as practically possible by the Project Coordinator.

vi. How will data and/or specimens be transported?

As noted above, most data from the research visits will be collected using laptop computers or tablets, reducing the need for paper or hard copy data. If rare instances when this information is necessary, paper and hard copy data will be transported as needed by pertinent study team members. Data obtained via home visits will be delivered to secure AU or UGA facilities as soon as practically possible. Electronic data will be shared among study team members using a secure internet connection and an approved data management software program that will be password protected.

8. Provisions to Monitor the Data to Ensure the Safety of Subjects

This study involves no more than minimal risk study and this section is not required. ☐ **N/A**

The plan might include establishing a data monitoring committee and a plan for reporting data monitoring committee findings to the IRB and the sponsor.

Data and Safety Monitoring Plan
Title: Responsive Parenting, Sleep, and Rapid Weight Gain among African American Infants
Grant # 1R01DK112874-01

I. Overview

Purpose of the Study:

The present study examines whether providing mothers of newborns with responsive parenting guidance during the first weeks of life to promote infant sleep and soothing can reduce rapid weight gain for African American infants born in low SES contexts, and also examines the risk and protective factors that may affect program efficacy.

Adherence Statement:

The Data and Safety Monitoring Plan (DSMP) outlined below for R01DK112874 will adhere to the protocol approved by the Augusta University IRB.

Overall Framework for Safety Monitoring and Information to be Monitored:

The data and safety monitoring plan (DSMP) for this intervention trial focuses on close monitoring of infant growth by the principal investigators (PIs) in conjunction with a Data Safety Monitor, along with prompt reporting of adverse events to the NIH/NIDDK and to the Institutional Review Board (IRB) at Augusta University. Because behavioral interventions aimed at reducing rapid weight gain could theoretically result in insufficient weight gain by study participants, infants' growth will

be closely monitored by the investigators, who will be overseen by the project's Safety Monitor, Dr. Reda Bassali. The decision to include a Data Safety Monitor instead of a Data and Safety Monitoring Board (as in the original proposal) was made in April 2018 in consultation with our Program Officer at NIDDK, Voula Osganian, M.D., based on NIDDK guidelines that a Data Safety Monitor is more appropriate for single-site clinical trials that are not masked and are minimal risk, as is the case in the current study.

II. Adverse Events

In this study, an adverse event shall be defined as any detrimental change in the participant's condition, whether it is related to the study interventions, study outcomes, or to another unrelated illness. Adverse effects may be (a) unrelated to the study interventions, (b) potentially related to the study interventions, or (c) related to the study interventions.

Adverse Events Unrelated to the Study Interventions:

Adverse events due to illnesses unrelated to study interventions may be grounds for withdrawal if the illness is considered significant by the study investigators or if the participant is no longer able to effectively participate in the study. A significant illness would be one that would compromise the child's ability to function normally and thrive, such as the diagnosis of a malignancy, illness characterized by growth problems, disease requiring ongoing and intensive treatment, and/or one requiring repeated hospitalizations or physician visits. Subjects experiencing routine, minor, self-limited acute illnesses that typically occur during infancy and do not affect long-term growth will not be recorded, and the child will continue to participate in the study. Examples of minor illnesses include acute otitis media, bronchiolitis, upper respiratory infections, urinary tract infections, and gastroenteritis. Medications for acute, self-limited illnesses such as those stated above will not be recorded, but chronic medication use (> 1 month) will be recorded.

Other adverse events that could affect growth include a milk-protein allergy, other food allergies, or physician-diagnosed gastroesophogeal reflux disease (GERD) requiring medication. Surgical conditions such as intestinal malrotation and pyloric stenosis also would impact infant feeding and weight gain. Therefore, these will be recorded and if the study investigators determine that these conditions can affect long-term growth, the participant will be withdrawn from the study.

Documentation of an adverse event unrelated to study interventions that are not considered minor illnesses of childhood and those that can significantly affect growth will be recorded using Augusta University's interactive digital form for reporting adverse events

Adverse Events Related or Potentially Related to the Study Interventions:

It is theoretically possible that behavioral interventions designed to prevent rapid weight gain during infancy could result in underfeeding by parents and insufficient growth. The NIH-funded studies that serve as the foundation for the current project did not find an association between study interventions and these adverse events. While the results of these previous projects are reassuring, weight status and growth will be closely monitored and evaluated at frequent intervals in the current project in order to monitor the study intervention for potential adverse events. In the current study, there are several ways a potential adverse event related to growth will be identified:

1. Weight-for-age below the 5th percentile using current CDC growth charts, based on WHO data from birth to 2 years.

2. Downward crossing of two major percentile lines on the WHO weight-for-age growth chart statistically evaluated as equal to or greater than a -0.67 Z-score change in order to provide a consistent measure across subjects.

Recognizing that growth monitoring is typically performed at all regularly scheduled primary care appointments, we will also ask mothers if their infant's PCP expressed concerns that the infant is demonstrating insufficient weight gain. In turn, the study team will determine if the infant meets one of these screening criteria above when assessed at study visits at 1 week, 3 weeks, 8 weeks, or 16 weeks.

During each study visit, home visit staff will assess weight. Within one week of the visit, the Project Coordinator will plot each child's growth on the CDC growth charts to allow for identification of potentially concerning growth patterns. Dr. Stansfield will be immediately alerted by the Project Coordinator to any concerning patterns (i.e., weight-for-age below the 5th percentile or downward crossing of two major percentile lines). After each child is closely evaluated, Dr. Stansfield will then contact the mothers by phone when necessary (i.e., when there is a potential adverse event related to infant growth), to discuss their infants' growth and whether a visit with the infant's primary care provider is necessary. If Dr. Stansfield is unable to reach the infant's mother and/or if the mother requests it, Dr. Stansfield will contact the infant's primary care provider directly. Dr. Stansfield will document these calls as they occur as a research note. Dr. Stansfield will review growth charts of all active study participants at least twice monthly.

For any individual child that meets initial screening criteria for growth concerns, numerous factors will be considered in determining whether the child's growth is problematic and/or related to study interventions. Examples of such factors include genetic potential based upon parental size, the participant's linear growth, feeding mode (breast milk vs. formula), and interval illnesses.

The study's informed consent document will include information indicating that the study team will communicate with the participant's PCP if necessary; all infants are required to have a PCP prior to discharge from the hospital and are expected to receive normal medical care (e.g., well-baby visits) throughout the study as is typical for infants. Doing so will allow for open lines of communication between the study team and the PCPs in the event of concerns related to growth. If either the primary care provider or the study investigators believe that it is possible that these growth patterns are a negative result from study participation, the child will be withdrawn from the study.

Expected Rates for Failing Screening Criteria for Adverse Events Potentially Related to Study Interventions:

Using standard population distributions, it is expected that 5% of children will be below the 5th percentile on the weight-for-age growth chart. For downward crossing of two major centile lines, it is expected that this will occur with some frequency given the well-established phenomenon of regression to the mean, which suggests that infants born at the higher percentiles have a higher probability of moving downward to the population mean. The results of our pilot study suggest that 15% of participants may experience this, and that will serve as the cut-off alarm value for this study. Combined, these two screening criteria allow for up to a 20% adverse event rate for those events related or potentially related to the study interventions.

Process by which Adverse Events Will be Managed and Reported:

As outlined above, we will continuously monitor adverse event rates in all participants. Dr. Stansfield will have primary responsibility for reporting Adverse Events, Serious Adverse Events, and Unanticipated Problems to the Augusta University IRB, the study's Safety Monitor, and the NIH/NIDDK as required. If Dr. Stansfield is unable to fulfill these duties, PI Lavner will have secondary responsibility for reporting.

a. Describe what data are reviewed, including safety data, untoward events, and efficacy data.

As described above, adverse events reports and infant growth will be monitored.

b. Describe how the safety information will be collected (e.g., with case report forms, at study visits, by telephone calls with participants).

We plan to collect adverse event data from the intervention and control groups as they occur using adverse event reports.

c. Describe the frequency of data collection, including when safety data collection starts.

The Project Coordinator will plot each child's growth on the CDC charts within one week following study visits to allow for identification of potentially concerning growth patterns. Dr. Stansfield will review the growth charts of active study participants at least twice monthly to allow for close monitoring and contact mothers as necessary. Safety reports will be sent to Dr. Stansfield, the PIs, and the Safety Monitor (Dr. Reda Bassali) twice per year. The Project Coordinator will be responsible for assembling the data and producing these reports in conjunction with the study statistical team, as well as assuring that all parties obtain copies of these reports.

d. Describe who will review the data.

Data will be reviewed as described above by Drs. Stansfield and Lavner, by the Project Coordinator, and by the Safety Monitor. The Safety Monitor for the study will be Reda Bassali, M.D., Associate Professor of Pediatrics at Augusta University. Dr. Bassali is Division Chief of the Department of General Pediatrics at Augusta University and has the necessary expertise and experience to serve as an objective Safety Monitor for this trial. Dr. Bassali is not involved in or affiliated with the grant in any capacity other than in his role as Safety Monitor. He has agreed to serve as the Safety Monitor for this project.

e. Describe the frequency or periodicity of review of cumulative data.

We will continuously monitor adverse event rates in all participants. PI Stansfield will have primary responsibility for reporting Adverse Events, Serious Adverse Events, and Unanticipated Problems to the AU IRB, the study's DSMB, and the NIH/NIDDK as required. PI Lavner will have secondary responsibility for reporting.

Safety reports will be sent to the study statistician, the PIs, and the Safety Monitor twice yearly. The Project Coordinator will be responsible for assembling the data and producing these reports in conjunction with the study statistical team, as well as assuring that all parties obtain copies of these reports.

f. Describe any conditions that trigger an immediate suspension of the research.

Stopping Rules:

As outlined above, we will continuously monitor adverse event rates in all participants. The study investigators, together with the Safety Monitor, will alert the IRB and the NIH/NIDDK if a greater

than or equal to 20% adverse event rate potentially due to study interventions should occur in the treatment group.

Any participant with a serious adverse event related to the study interventions will be discontinued from the study. Should insufficient weight gain be detected and the cause of this weight gain is related or potentially related to the study interventions as determined by Drs. Stansfield and Lavner, the participant will be discontinued from the study. Other non-minor concurrent illnesses or major changes in the family social environment would also lead to discontinuation as determined by the investigators.

As described in detail above, we will closely monitor rates of insufficient growth for individual infants and by study treatment group. In addition to individual level monitoring, the study statistician will analyze rates of insufficient growth by treatment group on a biannual basis if necessary. If there are significantly more children with inadequate growth in the intervention group at any point, these data will be presented to the Augusta University and UGA IRBs, the study's Safety Monitor, and the NIH/NIDDK so that a decision can be jointly made as to whether the trial needs to be suspended.

We acknowledge that there are other situations that could occur that might warrant stopping the trial, and these concerns will be discussed by Dr. Stansfield, Dr. Lavner, and the Safety Monitor as needed.

9. Withdrawal of Subjects ☐ N/A

a. *If applicable, describe anticipated circumstances under which subjects will be withdrawn from the research without their consent.*<br>Any participant with a physician-diagnosed serious adverse event related to the study interventions will be discontinued from the study. Should insufficient weight gain be detected and the cause of this weight gain is potentially related to the study interventions as determined by the PIs after consultation with the participant's primary care provider, the participant will be discontinued from the study. Other non-minor concurrent illnesses or major changes in the family social environment would also lead to discontinuation as determined by the investigators.	☐ N/A
b. *If applicable, describe any procedures for orderly termination.*<br>In the event that a subject is withdrawn from the research without their consent (whether due to unforeseen change in meeting inclusion criteria or an adverse event), all contact with subject will be ceased. Subjects will receive monetary compensation only for those appointments and study requirements met until the date of withdrawal. Any materials provided (bassinet, intervention materials) as part of the study procedures will remain with the subject. Only if agreed to at the time of consent, data collected for that subject dyad until the date of withdrawal will remain part of the study data and included in researcher analysis where possible.	☐ N/A
c. *If applicable, describe procedures that will be followed when subjects withdraw from the research, including partial withdrawal from procedures with continued data collection.*<br>In the event that subjects withdraw from the research, all contact with subject will be ceased. Subjects will receive monetary compensation only for those appointments and study requirements met until the date of withdrawal. Any materials provided (bassinet, intervention materials) as part of the study procedures will remain with the subject. Only if	☐ N/A

agreed to at the time of consent, data collected for that subject dyad until the date of withdrawal will remain part of the study data and included in researcher analysis where possible.	

10. Risks to Subjects

a. *List the reasonably foreseeable risks.*<br><br>*Potential risks*. All participants will continue to receive their standard care from their medical providers. Our previous research found no increased risk of adverse events, or specifically failure to thrive, among those infants randomized to receive the multi-component prevention program. Given that the proposed study is focused on infant sleep and soothing, rather than focused on feeding, and is of significantly shorter duration than our previous multi-component trials, there is extremely minimal risk of insufficient weight gain and/or failure to thrive in the experimental group.	
b. *If applicable, describe any costs that subjects may be responsible for because of participation in the research.*	■ N/A
c. *If applicable, describe risks to others who are not subjects.*	■ N/A

11. Potential Benefits to Subjects

Describe the potential benefits that individual subjects may experience from taking part in the research.

Participants may experience improved health outcomes such as improved sleep and soothing, and healthier weight status during the first 16 weeks of life. All participants may benefit from sleep safety and those in the safety control arm may benefit from the home-based safety interventions. Childhood obesity may be prevented in some subjects in the study. There may also be positive effects for mothers' individual and relationship functioning.

12. Confidentiality

Describe the procedures for maintenance of confidentiality.

Subject confidentiality will be a priority for the research team, with prudent measures taken to ensure security of data and personal information. Upon enrollment, subjects will be assigned a study ID number which will serve as a code associated with all collected data. This code will be linked to the subject in the project directory, which will be password protected using an approved data management software system. Only pertinent study team members (PIs, coordinators) will have access to any and all data using a secure internet connection. Electronic and paper versions of de-identified data will be stored separately from any files that may contain subject names.

13. Consent Process

If you are obtaining consent of subjects describe the consenting process.

At the time of recruitment (AU newborn nursery), qualified mothers will be asked to read and sign a document indicating informed consent for herself and a separate parental permission document indicating consent for her infant to participate. The approved consent/permission documents will detail all study procedures and address incentives, risks, benefits, confidentiality and potential withdrawal. Mothers will have the opportunity to have any questions answered by the recruitment coordinator or PI prior to making the decision to enroll.

14. Compensation for Research-Related Injury

This section is not required when research involves no more than Minimal Risk to subjects. ☒ **N/A**

a. *Describe the available compensation in the event of research related injury.*

15. Resources Available ☐ N/A

a. *Describe the availability of medical or psychological resources that subjects might need as a result of an anticipated consequences of the human research.*

Although we do not anticipate any negative consequences of this research, all participants will receive a handout at the first visit with a list of community resources.

b. *Describe your process to ensure that all persons assisting with the research are adequately informed about the protocol, the research procedures, and their duties and functions.*

All study team members will be trained according to individual responsibilities with respect to the project and managed by the PIs and the Project Coordinator. Regular meetings of the core study team from each site (AU and UGA) will ensure efficient communication and management of personnel. All study team members will maintain appropriate training certifications.

Consistent with protocols used in the pilot trial and efficacious programs for rural African American families, the Community Research Associates (CRAs) who will serve as the Sleep-SAAF intervention home visit facilitators are African American community members from communities similar to the ones where the intervention will be implemented. CRAs must have a minimum education level of high school graduation and are selected based on information from interviews and references that attests to their communication skills, engaging personalities, and ability to implement a structured program with fidelity. CRAs will receive training in the implementation of curriculum activities, guided practice in delivering and pacing curriculum segments, and facilitator self-care. They will work from a detailed manual that describes all facets of program delivery. Didactic material, role-playing exercises, and modeling will be used to teach the protocol for each session. To ensure ongoing quality control and fidelity, CRAs will meet with the Project Coordinator and/or Intervention Coordinator regularly.
