# Supplementary material for: Effects of a Responsive Parenting Intervention Among Black Families on Infant Sleep: A Secondary Analysis of the Sleep SAAF Randomized Clinical Trial
Source: JAMA Netw Open. 2023 Mar 31;6(3):e236276. doi: 10.1001/jamanetworkopen.2023.6276 (PMC10066466; doi:10.1001/jamanetworkopen.2023.6276)
Supplement: Supplement 2. — eMethods. eTable. Sleep SAAF 3 week sleep variables by study group eReferences. [file jamanetwopen-e236276-s002.pdf]

Supplemental Online Content

Lavner JA, Hohman EE, Beach SRH, Stansfield BK, Savage JS. Effects of a responsive parenting intervention among Black families on infant sleep: a secondary analysis of the Sleep SAAF randomized clinical trial. *JAMA Netw Open*. 2023;6(3):e236276. doi:10.1001/jamanetworkopen.2023.6276

eMethods.
eTable. Sleep SAAF Week 3 Sleep Variables by Study Group
eReferences.

This supplemental material has been provided by the authors to give readers additional information about their work.

© 2023 Lavner JA et al. *JAMA Network Open.*

eMethods

Brief Infant Sleep Questionnaire (BISQ)[1]

Note: Items highlighted in yellow were asked at 16 weeks only. Items highlighted in blue were asked at 3 and 8 weeks only. All others collected at 3, 8, and 16 weeks.

1. How many hours does your baby spend sleeping during the NIGHT (between 7:00 in the evening and 8:00 in the morning)?

1a: Hours _______

1b: Minutes _______

2. How many hours does your baby spend sleeping during the DAY (between 8:00 in the morning and 7:00 in the evening)?

2a: Hours _______

2b: Minutes _________

3. Where does your baby sleep most of the time?

1	2	3	4
In baby's own room	In parent's room	In sibling's or other room	Other: (specify)

3a: Please specify your selection: ______________

4. Which of the following does your baby sleep in most of the time?

1	2	3	4	5	6
Bassinet/baby box	Infant seat	Swing	Crib	Parent's bed	Other: (specify)

4a: Please specify your selection: _______________

5. Do you have a bedtime routine for your baby?

1	2
No	Yes

6. What time do you usually start your baby's bedtime routine?

1. 5:00PM	7. 6:30PM	13. 8:00PM	19. 9:30PM	25. 11:00PM
2. 5:15PM	8. 6:45PM	14. 8:15PM	20. 9:45PM	26. 11:15PM
3. 5:30PM	9. 7:00PM	15. 8:30PM	21. 10:00PM	27. 11:30PM
4. 5:45PM	10. 7:15PM	16. 8:45PM	22. 10:15PM	28. 11:45PM

© 2023 Lavner JA et al. *JAMA Network Open*.

5. 6:00PM	11. 7:30PM	17. 9:00PM	23. 10:30PM	29. 12:00AM
6. 6:15PM	12. 7:45PM	18. 9:15PM	24. 10:45PM	

7. What is the **LAST** thing you usually do with your baby before you put him/her to bed?

1. Bath	5. Listen to music	9. Swaddle	13. Say prayers
2. Massage	6. White noise	10. Give pacifier	14. Sing songs
3. Read books	7. Watch television	11. Play	15. Put in a swing
4. Rock baby	8. Give a bottle, drink, or nurse	12. Cuddle	16. Other (specify):

7a: Please specify your selection: ____________

8. What time do you usually put your baby to bed at night? (time of turning out the light)

1. 5:00PM	7. 6:30PM	13. 8:00PM	19. 9:30PM	25. 11:00PM
2. 5:15PM	8. 6:45PM	14. 8:15PM	20. 9:45PM	26. 11:15PM
3. 5:30PM	9. 7:00PM	15. 8:30PM	21. 10:00PM	27. 11:30PM
4. 5:45PM	10. 7:15PM	16. 8:45PM	22. 10:15PM	28. 11:45PM
5. 6:00PM	11. 7:30PM	17. 9:00PM	23. 10:30PM	29. 12:00AM
6. 6:15PM	12. 7:45PM	18. 9:15PM	24. 10:45PM	

9. In the past 7 days, bedtime was difficult for my baby (Example: fussing, crying).

1	2	3	4	5
Never	Almost never	Sometimes	Almost always	Always

10. In the past 7 days, my baby had a difficult time falling asleep at night.

1	2	3	4	5
Never	Almost never	Sometimes	Almost always	Always

11. How does your baby typically fall asleep at night? (check all that apply)

a. While being bottle fed

b. While being breastfed/nursing

c. While being rocked

d. While being held

© 2023 Lavner JA et al. *JAMA Network Open.*

e. Swaddled

f. Sucking thumb/finger

g. While using a pacifier

h. While listening to white noise

i. Other (specify):

11a: Please specify your selection: ________________________

12. How long does it typically take your baby to fall asleep when put down for nighttime sleep? (Example: If you put your baby to bed at 8:15 PM and your baby fell asleep at 8:30 PM, it took 15 minutes for your baby to fall asleep). Minutes **(**Open Ended)

13. I put my baby down to sleep…

1	2
On his/her tummy	On his/her back

14. How often do you put your baby to bed while he/she is still awake?

1	2	3	4
Never	Sometimes	Usually	Always

15. How many times does your baby typically wake during the night?

0	1	2	3	4	5
0 times per night	1 times per night	2 times per night	3 times per night	4 times per night	5 or more times per night

16. In the past 7 days, my baby woke up during the night and had trouble falling back asleep.

1	2	3	4
Never	Sometimes	Usually	Always

17. On average, when your baby wakes during the night, how long is he/she awake?

1	2	3	4	5
0-15 minutes	15-30 minutes	30-60 minutes	1-2 hours	More than 2 hours

18. How many times is your baby fed after waking during the night?

0	1	2	3	4	5
0 times per night	1 times per night	2 times per night	3 times per night	4 times per night	5 or more times per night

© 2023 Lavner JA et al. *JAMA Network Open.*

19. When your baby wakes up during the night, what do you do? (check all that apply)

a. Pick up my baby and hold/rock him/her until he/she falls asleep

b. Pick up my baby and put him/her back down while he/she is awake

c. Rub or pat my baby but do not pick up or take out of crib/bed

d. Feed baby back to sleep

e. Feed baby and put down while baby is awake

f. Give my baby a pacifier

g. Change diaper

h. Comfort my baby verbally but don't pick him/her up or take him/her out of crib/bed

i. Bring my baby to bed

j. Let my baby cry and fall back to sleep by himself/herself

k. Give my baby a few minutes to see if he/she falls back to sleep

l. Play with my baby until he/she is ready to go back to sleep

m. Watch television or a video with my baby until he/she falls asleep

n. Sing to baby

o. N/A

p. Other, please specify:

q: Please specify your selection: ______________________

20. On a typical night, what is the longest stretch of time that your baby stayed asleep during the night (between 7 pm and 7 am) without waking up?

20a: Hours _________

20b: Minutes __________

21. During the past week, how difficult was it to put your baby down for bedtime at night?

1	2	3	4
Very difficult	Somewhat difficult	Somewhat easy	Very easy

22. Overall, how well does your baby usually sleep at night?

1	2	3	4
Very well	Well	Poorly	Very poorly

© 2023 Lavner JA et al. *JAMA Network Open.*

23. Do you wake your baby to feed him/her before you go to bed? (Sometimes called a dream feed)

1	2
Yes	No

24. What time does your baby typically wake up in the morning?

1. 5:00AM	7. 6:30AM	13. 8:00AM	19. 9:30AM	25. 11:00AM
2. 5:15AM	8. 6:45AM	14. 8:15AM	20. 9:45AM	26. 11:15AM
3. 5:30AM	9. 7:00AM	15. 8:30AM	21. 10:00AM	27. 11:30AM
4. 5:45AM	10. 7:15AM	16. 8:45AM	22. 10:15AM	28. 11:45AM
5. 6:00AM	11. 7:30AM	17. 9:00AM	23. 10:30AM	29. 12:00PM
6. 6:15AM	12. 7:45AM	18. 9:15AM	24. 10:45AM	

25. How many naps does your baby take on a typical day?

1	2	3	4	5	6	7	8
0 - my baby does not nap	1	2	3	4	5	6	More than 6

26. On average, how long does your baby sleep when put down for a nap?

1	2	3	4	5
0-30 minutes	30-60 minutes	60-90 minutes	90-120 minutes	More than 120 minutes

27. How many days per week does your baby take a daytime nap?

1	2	3	4
6-7 days per week	4-5 days per week	2-3 days per week	1 day per week

© 2023 Lavner JA et al. *JAMA Network Open.*

SSP: Safe Sleep Practices[2]

Asked at 16 weeks only

1. How often does your baby sleep in his/her own crib/bassinet, including for naps?

1	2	3	4	5
Always	Usually	Sometimes	Occasionally	Never

2. How often does your baby sleep with toys/stuffed animals in his/her crib?

1	2	3	4	5
Always	Usually	Sometimes	Occasionally	Never

3. How often does your baby sleep in the same bed as you, another adult, or another child?

1	2	3	4	5
Always	Usually	Sometimes	Occasionally	Never

4. Do you, or anyone else in the household, smoke?

1	2
Yes	No

5. How often do you place your baby on his/her back for sleep, including naps?

1	2	3	4	5
Always	Usually	Sometimes	Occasionally	Never

6. How often does your baby sleep on a sofa/couch, waterbed, or soft mattress?

1	2	3	4	5
Always	Usually	Sometimes	Occasionally	Never

© 2023 Lavner JA et al. *JAMA Network Open.*

eTable. Sleep SAAF Week 3 Sleep Variables by Study Group

	3 Weeks	
	**RP (n=102)**	**Control (n=101)**
Time in bed (minutes), mean (SD)	563 (124)[b]	574 (102)[c]
Has a bedtime routine, n (%)	52 (51.0)	59 (58.4)
Fed as last activity before bed, n (%)	70 (68.6)	70 (69.3)
Bedtime 8pm or earlier, n (%)	17 (17.7)[b]	26 (27.1)[b]
Falls asleep swaddled, n (%)	13 (12.8)	14 (13.9)
Falls asleep with pacifier, n (%)	20 (19.6)	23 (22.8)
Falls asleep with white noise, n (%)	5 (4.9)	4 (4.0)
Falls asleep being held, n (%)	36 (35.3)	39 (38.6)
Dream feeds, n (%)	55 (55.0)[d]	46 (46.0)[d]
Usually/always put to bed awake, n (%)[a]	9 (8.8)	7 (6.9)
Put to sleep on back, n (%)	101 (99.0)	100 (99.0)
Usually sleeps in own crib/bassinet, n (%)	83 (82.2)[e]	89 (88.1)
Night wakings (times per night), mean (SD)	2.8 (1.0)	2.6 (1.0)
Night feedings (times per night), mean (SD)	2.4 (0.9)	2.4 (0.9)[f]
Response to infant night wakings, n (%)		
Give few minutes to fall back asleep	20 (19.6)	26 (25.7)
Pick up and hold/rock back to sleep	59 (57.8)	52 (51.5)
Rub/pat but do not pick up	8 (7.8)	7 (6.9)
Feed back to sleep	58 (49.6)	59 (58.4)
Feed but put back down awake	15 (14.7)	8 (7.9)
Give pacifier	28 (27.5)	30 (29.7)
Bring to parent bed	2 (2.0)	6 (5.9)
Play with baby until ready to go back to sleep	16 (15.7)	22 (21.8)
Watch TV or video with baby until falls asleep	10 (9.8)	19 (18.8)
Sing to baby	17 (16.7)	16 (15.8)

[a]Response options were never, sometimes, usually, or always; [b]N=96; [c]N=94; [d]N=100; [e]N=101; [f]N=99.

© 2023 Lavner JA et al. *JAMA Network Open.*

eReferences

1. Sadeh A. A brief screening questionnaire for infant sleep problems: Validation and findings for an Internet sample. *Pediatrics*. 2004;113(6):e570-e577. doi:10.1542/peds.113.6.e570

2. Fowler AJ, Evans PW, Etchegaray JM, Ottenbacher A, Arnold C. Safe sleep practices and sudden infant death syndrome risk reduction: NICU and well-baby nursery graduates. *Clinical Pediatrics*. 2013;52(11):1044-1053. doi:10.1177/0009922813506038

© 2023 Lavner JA et al. *JAMA Network Open*.
