# Supplementary material for: Effects of a Responsive Parenting Intervention Among Black Families on Infant Sleep: A Secondary Analysis of the Sleep SAAF Randomized Clinical Trial
Source: JAMA Netw Open. 2023 Mar 31;6(3):e236276. doi: 10.1001/jamanetworkopen.2023.6276 (PMC10066466; doi:10.1001/jamanetworkopen.2023.6276)
Supplement: Supplement 3. — Data Sharing Statement [file jamanetwopen-e236276-s003.pdf]

Data Sharing Statement

Lavner. Effects of a Responsive Parenting Intervention Among Black Families on Infant Sleep. *JAMA Netw Open*. Published March 31, 2023. doi:10.1001/jamanetworkopen.2023.6276

Data
Data available: Yes
Data types: Deidentified participant data, Data dictionary
How to access data: Request for data should be sent to lavner@uga.edu.
When available: With publication

Supporting Documents
Document types: Informed consent form, Other (please specify)
Additional Information: Trial protocol
How to access documents: https://link.springer.com/article/10.1186/s12887-019-1583-7
When available: With publication

Additional Information
Who can access the data: Researchers whose proposed use of the data has been approved.
Types of analyses: To replicate the analyses reported in the article.
Mechanisms of data availability: With a signed data access agreement.
